# Enhancing COVID-19 classification of X-ray images with hybrid deep transfer learning models

**DOI:** 10.3389/frai.2025.1646743

**Published:** 2025-10-13

**Authors:** Maliki Moustapha, Murat Tasyurek, Celal Ozturk

**Affiliations:** ^1^Graduate School of Applied Science and Technology, Department of Computer Engineering, Erciyes University, Kayseri, Türkiye; ^2^Department of Computer Engineering, Kayseri University, Kayseri, Türkiye; ^3^Department of Software Engineering, Erciyes University, Kayseri, Türkiye

**Keywords:** artificial intelligence, deep transfer learning, image classification, k-fold, genetic algorithm

## Abstract

Deep learning, a subset of artificial intelligence, has made remarkable strides in computer vision, particularly in addressing challenges related to medical images. Deep transfer learning (DTL), one of the techniques of deep learning, has emerged as a pivotal technique in medical image analysis, including studies related to COVID-19 detection and classification. Our paper proposes an alternative DTL framework for classifying COVID-19 x-ray images in this context. Unlike prior studies, our approach integrates three distinct experimentation processes using pre-trained models: AlexNet, EfficientNetB1, ResNet18, and VGG16. Furthermore, we explore the application of YOLOV4, traditionally used in object detection tasks, to COVID-19 feature detection. Our methodology involves three different experiments: manual hyperparameter selection, k-fold retraining based on performance metrics, and the implementation of a genetic algorithm for hyperparameter optimization. The first involves training the models with manually selected hyperparameter sets (learning rate, batch size, and epoch). The second approach employs k-fold cross-validation to retrain the models based on the best-performing hyperparameter set. The third employed a genetic algorithm (GA) to automatically determine optimal hyperparameter values, selecting the model with the best performance on our dataset. We tested a Kaggle dataset with more than 5,000 samples and found ResNet18 to be the best model based on genetic algorithm-based hyperparameter selection. We also tested the proposed framework process on another separate public dataset and simulated adversarial attacks to ensure its robustness and dependability. The study outcomes had an accuracy of 99.57%, an F1-score of 99.50%, a precision of 99.44%, and an average AUC of 99.89 for each class. This study underscores the effectiveness of our proposed model, positioning it as a cutting-edge solution in COVID-19 x-ray image classification. Furthermore, the proposed study has the potential to achieve automatic predictions through the use of input images in a simulated web app. This would provide an essential supplement for imaging diagnosis in remote areas with scarce medical resources and help in training junior doctors to perform imaging diagnosis.

## 1 Introduction

The virus, SARS-CoV-2, also known as COVID-19, emerged in Wuhan, China, in late 2019 and caused unprecedented challenges worldwide, leading to a global pandemic with millions of affected and dead people despite uncounted efforts to restrain its impact through various socio-political and financial measures (Hageman, 2020; Elibol, 2021). In the first half of 2022, the World Health Organization (WHO) reported over 6.2 million deaths and 516 million diagnosed cases worldwide. Similar to the category of Severe Acute Respiratory Syndrome (SARS) and Middle East Respiratory Syndrome (MERS) (2), shortness of breath, fever, coughing, pneumonia, and respiratory distress are registered as its common symptoms. The virus's dangerous effects on communities and fast transmission among people accentuated the imperative need for stringent measures. Almost all governments worldwide have implemented safety protocols, including social distancing, to control the spread of the pandemic. Despite the application of the protocols, the scale of infections and limitations at the hospital level underscored the mortality risk associated with delayed detection and progressive respiratory failure (Xu Z. et al., 2020). This issue increased the demand for effective and practical medical research and diagnosis solutions because precisely and quickly recognizing COVID-19 is a critical step in controlling the widespread disease (Jin et al., 2020). During the pandemic, Reverse Transcription Polymerase Chain Reaction (RT-PCR), one of the most used gold standards for COVID-19 diagnosis, faced challenges due to its time-consuming nature and susceptibility to a high false-negative rate (Xiao et al., 2020; Arevalo-Rodriguez et al., 2020). To overcome similar challenges, computed tomography (CT) and X-ray image analysis have shown promise in detecting lung diseases such as pneumonia, tuberculosis, and COVID-19 (Ismael and Şengür, 2021). However, the need for more specialized human resources, especially in poorer regions, hinders the widespread adoption of these imaging technologies. The scientific community has turned to computer-aided intelligent decision-making systems to automate the necessary processes to overcome this challenge. From these perspectives, advanced artificial intelligence (AI) techniques, particularly deep learning, have emerged as paramount tools for addressing the shortcomings of traditional diagnostic methods. This study contributes to the previously stated research efforts in the literature for COVID-19 virus detection and classification by using deep transfer learning techniques (DTL) with possible integration of heuristic algorithms like genetic algorithms to accurately classify COVID-19 from chest X-ray pictures. This research aspires to overcome the limitations of time-consuming issues of hyperparameter setting and resource-intensive utilization of traditional image detection models and experiments by employing different training processes. The advantage of the transfer learning approach is that it offers a flexible and scalable general solution for COVID-19 detection in healthcare systems.

Based on our review of related studies, several studies have used deep learning models such as EfficientNet, AlexNet, VGG16, ResNet18, and others to diagnose lung disease from chest CT or X-ray images (Marefat et al., 2023; Sultana et al., 2023). However, studies have yet to use another training process for DTL using k-folds or fine-tuning the pre-trained network hyperparameters using heuristic methods like genetic algorithms (GA) and specifically compare the performance of these models for COVID-19 data classification in detail. Therefore, this is one of the knowledge gaps this study bridges.

This study is the first to compare in more detail the performance of these pre-trained models based on all performance evaluation metrics for COVID-19 data classification using a benchmark chest X-ray image dataset. It presents a comprehensive and in-depth performance comparison of these models with some modifications and optimisations of hyperparameters applied to improve their performances.

The paper is organized as follows: The subsequent section (section two) examines prior and recent research in the literature related to deep learning implementation and other hybrid methods for COVID-19 detection and classification. Section three describes the proposed framework with the newly implemented methodology using the transfer learning technique, including the descriptions of the pre-trained models used in this research. The illustrations of experimental processes are depicted in section four, and the evaluated results are discussed in section five, followed by a detailed comparison with relevant related works, including the limitation of this study in section six. The last section concludes the paper with some suggestions for future study.

### 1.1 Contributions

The significant contributions of this study can be outlined as follows:

- Unlike other studies, this research achieved a more detailed comparison of the performance of several pre-trained models based on various evaluation metrics for COVID-19 data classification using publicly available chest X-ray images from the Kaggle database.
- This study developed and improved distinct pre-trained deep learning models on COVID-19-related X-ray image classification using the transfer learning strategy integrated with the k-fold process.
- It also performs the hyperparameter tuning using the genetic algorithm to facilitate the near-optimal hyperparameter value determination, leading to the best model performances.
- Based on all the possible evaluation metrics (training, validation, and testing accuracy, F1-score, precision, recall, AUC, inference execution time), instead of only accuracy like performed in other works, the best model (the ResNet-based) of this study and the remaining models are compared with recent state of the art models.
- The proposed study has the potential to achieve automatic predictions through the use of input images in a simulated web app; therefore, it can serve as an essential supplement for imaging diagnosis in remote areas with scarce medical resources and help in training junior doctors to perform imaging diagnosis.

### 1.2 Contributions

The significant contributions of this study are as follows:

- **Hybrid multi-phase evaluation framework:** the study proposes a three-tiered experimental design that integrates manual tuning, k-fold cross-validation, and genetic algorithm (GA)-based hyperparameter optimization, applied across four established architectures. This systematic approach provides a deeper and replicable analysis of the impact of various tuning strategies on model performance, addressing a gap that has not been comprehensively explored in prior studies.
- **Cross-dataset robustness and deployment simulation:** beyond using publicly available Kaggle data, we evaluate models on an external dataset and under simulated adversarial conditions to assess robustness. Furthermore, we design a proof-of-concept web-based diagnostic aid, demonstrating potential utility in remote or resource-constrained healthcare environments.
- **Comprehensive evaluation across metrics:** unlike many previous works that rely solely on accuracy, we report training, validation, and testing performance across multiple metrics (F1-score, precision, recall, AUC, and inference time), enabling a more holistic and fair comparison with recent state-of-the-art models.

### 1.3 Scope and outline

This study focuses on applying the deep transfer learning method to develop models capable of detecting or classifying X-ray scans as COVID-19 or normal cases.

## 2 Related studies

According to the literature review, different AI and machine learning techniques have solved various healthcare-related problems. Deep learning, a subdomain of machine learning, has been used as an approach adopted by many researchers to categorize the coronavirus in X-rays and CT images. This section provides a comprehensive overview of recent existing studies that have used pre-trained models (and possibly with machine learning algorithms) for diagnosing lung diseases, especially COVID-19, from chest X-ray images.

(Jangam et al. 2022) presented a new stacked ensemble that detects COVID-19 from individuals either from their CT scans or X-rays. Their stacked model was developed using four different and heterogenous models, the VGG19, Rest101, DenseNet169, and WideRestNet502. They designed a new weighted average model using the best-performing selected models based on each model's performance. Their model outputs uniformly good performance on five different datasets. An alternative deep-learning approach for detecting COVID-19 was introduced in the study by (Chakraborty et al. 2022). Like (Aggarwal et al. 2022) work, their model classifies images into three classes. Despite using a larger dataset of 10,040 samples with some imbalance issues, the authors handled sample size issues with data augmentation and image resizing techniques. Notably, pre-trained models like AlexNet, VGG, ResNet, and DenseNet were used for performance comparison even though their work methodology needed to specify the fine-tuning process for these networks. The research's strength was performing image segmentation to focus on the relevant image area, improving training quality and model results. Their experiments consisted of 200 training epochs, 10% validation, and 20% testing with a batch size of 50 and considered the confusion matrix, accuracy, F1-score, precision, recall, and the ROC curve for evaluation metrics. The proposed model reached 96.43% detection accuracy and 93.68% sensitivity. However, even with augmentation, the dataset is assumed insufficient, and more information on hyperparameter comparison and network fine-tuning details could be mentioned. (Yadlapalli et al. 2022) investigated the classification of CT scans to diagnose COVID-19 patients. Their method has adopted K-means clustering for image segmentation, separating the Area of interest from the background, as a new technique in the field. The segmented images were provided into the pre-trained VGG16 network, and a three-layer CNN was also created from scratch. While image segmentation made learning more accessible for both models, it also raised some overfitting by decreasing the number of features in an image. Both models have been experimented on a COVID-19-CT dataset containing 349 positive and 397 negative scans. However, VGG16 outperformed other networks with the highest results accuracy of 89%, an F1-Score of 88%, and an AUC of 0.94. Other metrics, such as precision, recall, and sensitivity, were not specified.

Another work in the deep learning application field is that (Khan et al. 2022) achieved by comparing the performance of three different pre-trained networks based on two strategies, incorporating regularization techniques to improve COVID-19 detection. The 21,165 image size of the dataset used was collected from Github and Kaggle and divided into four categories: normal, lung opacity, viral pneumonia, and COVID-19. Furthermore, admitting the inequality in the dataset, data augmentation techniques such as rotation and horizontal and vertical flipping were used to manage the issue partially, estimating that more data would be an additional influential solution. The images were rescaled and resized as part of the preprocessing steps. EfficientNetB1 surpassed other models in a comparison that implicated fine-tuning hyperparameters like dropout percentage and learning rate, with strategy II showing superior performance, although precise details were not provided. The top-performing model achieved an accuracy of 96.13%, evaluated using metrics including the confusion matrix, accuracy, F1-score, precision, and recall.

Different from other studies, a comparative study involving eight fine-tuned pre-trained architectures for the classification of X-ray images into various disease types was accomplished by (Aggarwal et al. 2022). Two datasets were used: one set was composed of normal and COVID-19 with 709 samples, and the other comprised only pneumonia, subdivided into bacterial and viral subtypes. Due to their small sizes, the datasets were divided into 80% for training and 20% for testing during the experimentations. Some data augmentation techniques were implemented to increase sample size and enhance model generalization. Contrast Limited Adaptive Histogram Equalization (CLAHE), image scaling, and resizing were all part of their preprocessing steps. DenseNet121 exhibited the highest accuracy (97%) for the first dataset, while MobileNetV2 achieved the best accuracy (81%) for the second dataset according to the evaluation metrics such as confusion matrix, accuracy, F1-score, precision, and recall. However, despite data augmentation, the article highlighted limitations, particularly the need for more samples. The study revealed weaknesses in F1-score results for bacterial and viral pneumonia, suggesting misclassifications, mainly into the COVID-19 category. The article needed more discussion on the fine-tuning architecture of networks and experimentation with hyperparameters, highlighting potential areas for improvement in comparative research.

With total images comprised of 1,125 images, 125 for COVID-19, 500 for pneumonia and 500 for No-Findings input, a deep learning model called DarkCovidNet was developed by (Ozturk et al. 2020) for COVID-19 detection. The study succeeded with 98.08% and 87.02% for binary and three classes.

A new combined model approach consisting of different phases was proposed by (Ozcan 2021) for COVID-19 detection in X-ray images using in-depth features. The research methodology comprises two main variants. The first one is single layer-based (SLB), and the other is future fusion-based (FFB) with a feature extraction mechanism. The FFB3 accomplished 99.52 % accuracy, better than the DarkNet accuracy (98.02 %). Another learning model was used by (Hemdan et al. 2020) to analyze individuals' X-rays and presented a COVIDX-Net model containing seven neural networks. (Wang et al. 2020) developed the COVID-Net model for COVID19 detection. Their presented deep model obtained 92.4% accuracy in many classes classification such as normal, non-COVID pneumonia. Medical experts have found that COVID-19 shares symptoms with pneumonia, including difficulties in breathing and chest heaviness. This similarity poses a challenge in distinguishing COVID-19 from other chest diseases. As an improved method different from single-class COVID-19 detection studies in the literature, (Rehman et al. 2021) suggested a framework capable of detecting 15 types of chest diseases, including COVID-19, using chest X-ray modality. Their suggested framework performed the classification task in two ways. The first way uses a deep learning-based CNN architecture with a soft-max classifier, while the other extracts deep features from the CNN's fully connected layer using a transfer learning strategy (Ozturk et al., 2023; Terzi and Azginoglu, 2023). Their approach enhances COVID-19 detection accuracy and increases prediction rates for different chest disorders. Approximated to other state-of-the-art models for diagnosing COVID-19 and other chest disorders, the experimental results prove that the proposed framework performed appreciatively. Mathematical models and optimization strategies, like hybrid methods combining deep learning with heuristic approaches, have been proven to play a crucial role in helping researchers provide a methodical framework to investigate complex biological systems and healthcare data. Within the same perspective, the study achieved by (Saeed et al. 2022) introduced a novel mathematical framework called Complex Fuzzy Hypersoft (CFHS) set for diagnosing and treating medical conditions. The CFHS set was designed to integrate the concepts of Complex Fuzzy (CF) and Hypersoft set, addressing uncertainty, ambivalence, and mediocrity in data through the incorporation of amplitude term (A-term) and phase term (P-term) of complex numbers simultaneously. The framework extends membership function values to the unit circle on an Argand plane, assuming the periodic character of data with the additional P-term. It divides various properties into sub-valued sets, allowing for a more comprehensive understanding. The framework extends membership function values to the unit circle on an Argand plane, considering the periodic character of data with the additional P-term. It divides various properties into sub-valued sets, allowing for a more comprehensive comprehension. As The CFHS framework set and its mapping and inverse mapping (INM) are designed to address various problems, their study ascertained the proposed concept by demonstrating a link between COVID-19 symptoms and medications. Thus, a distinct COVID-19 category was determined utilizing fuzzy intervals and CFHS mapping for disease identification and optimal medication selection. Furthermore, a generalized mathematical CFHS-mapping framework has been proposed to extract patient health information and forecast recovery time from COVID-19, and the overall outcomes of their work implementation prove to be effective for medical applications, especially in COVID-19 diagnosis, treatment, and optimize general health system functioning. Mask detection was another target domain of interest in CNN model implementation. However, considering other essential challenges related to this field, more advanced artificial intelligence techniques, such as cooperative agents, can be helpful. Thus, (Allioui et al. 2022) developed a novel multi-agent-based deep reinforcement learning (DRL) to underrate the long-term manual mask extraction and to enhance medical image segmentation frameworks. An altered version of the Deep Q-Network was engaged in their study to stimulate the mask detector to select masks from the image analyzed. The DRL has experimented on COVID-19 CT images to extract visual features from infected areas, enabling accurate clinical diagnosis and demonstrating its performance. This approach optimizes pathogenic diagnostic tests and saves time. Their testing phases utilized CT images from various cases (normal, pneumonia, viral, and COVID-19), and experimental validation achieved a precision of 97.12%, sensitivity of 79.97%, and specificity of 99.48%. The results indicate the significance of using DRL to extract CT masks for accurate COVID-19 diagnosis.

Assuming that, despite vast global research proposed on the COVID-19 pandemic, developing reliable and fast prediction mechanisms that precisely distinguish this infectious disease from other respiratory conditions remains a significant challenge, and the widely utilized clinical RT-PCR test encounters limitations, particularly in areas with limited testing facilities, due to its slow response time. Recent efforts emphasized using digital chest X-ray and CT scan images, utilizing deep transfer learning and ensemble methods with base classifiers. Although enhanced accuracy, ensembles often need improvement with computational intensity and slow prediction times. (Misra et al. 2023) introduced a parallel ensemble transfer learning-based Framework for COVID-19 named PETLFC for multi-class classification to address the challenge. Their study used three pre-trained models named VGG16, ResNet18, and DenseNet121 as backbone by fine-tuning them for a parallelized bagging-based ensemble for COVID-19 case prediction. The data parallel model is implemented on the PARAM SHAVAK HPC system on a dataset composed of 21,165 chest X-ray images (10,192 normal, 1,345 pneumonia, 3,616 COVID-19, and 6,012 lung opacity). Their presented PETLFC approach proves outstanding performance in accuracy and efficiency compared to state-of-the-art sequential ensemble approaches.

In order to solve challenges caused by the severe impact of the COVID-19 pandemic, such as a shortage of resources, including limited test kits, (Ayalew et al. 2022) presented a paper introducing a novel detection and classification approach named DCCNet, utilizing chest X-ray images for rapid COVID-19 diagnosis. Their suggested method merges a CNN with a histogram of oriented gradients (HOG) to assist in diagnosing COVID-19 for experts. The study assesses the diagnostic performance of the hybrid CNN model and the HOG-based method using chest X-ray images from the University of Gondar and online databases. They summarized that the evaluation results show impressive accuracy: DCCNet achieved 99.9% training and 98.3% test accuracy, and the HOG method reached 100% training and 98.5% test accuracy. The hybrid model outperforms existing models with 99.97% and 99.67% training and testing accuracy, surpassing state-of-the-art models by 6.7%, highlighting DCCNet's effectiveness in enhancing COVID-19 detection and classification in medical imaging. As country-related research, (Indumathi et al. 2022) published a paper that introduced machine learning (ML) algorithms for forecasting the current status of COVID-19 in the Virudhunagar district (India), categorizing affected regions into danger, moderate, and safe zones. By using the available COVID-19 dataset from March to July 2020. they reported that their deep learning algorithm achieved 98.06% accuracy, outperforming the C5.0 algorithm with an accuracy of 95.92%. Though details of the evaluation metrics have been specified, they conveyed that their proposed system enables health departments to swiftly predict danger zones and take prompt preventive actions against infections in different areas. In a similar vision of integrating machine learning algorithms with CNN for COVID-19 classification problems, another study conducted by (Salau 2021) suggested a practical method for classifying chest X-ray images as Normal or COVID-19 infected. They obtained images using open-source software and transmitted them through CNN layers (Max pooling, ReLU, dense). Later, SVM was used to classify images into predefined classes (COVID-19 or Normal), leveraging knowledge from the learning model. Their findings reveal encouraging results for all models, mainly augmentation, image cropping, and segmentation, which achieved efficient results with a training accuracy of 99.8% and a test accuracy of 99.1%. Assuming that despite different studies showing clinical decisions for COVID-19 on diagnosis and few studies have focused on clinical decisions for COVID-19, (Deriba et al. 2023) presented research intended to detect a coronavirus patient path based on the virus' biological traits and offered an adequate mechanism for the efficient decision support system that assists doctors in predicting a COVID-19 patient. Their model was trained and tested using data from 311 patients (69% male, 31% female) collected from three Ethiopian Hospitals between November 2021 and March 2022, with patients aged between 21 and 67, then used three Machine Learning (ML) algorithms, namely Nave Bayes (NB), Artificial Neural Network (ANN), and Support Vector Machine (SVM) for experimentation. According to evaluation metrics, the ANN achieved 97% for recall, 96% for precision, and 98.3% for F1-score, demonstrating that ANN performed better than the NB classifier by 8.3% on average and better than SVM by 4.75%. (Wubineh et al. 2023) presented a paper suggesting an expert system to analyze the disease effectively diagnosing COVID-19 based on its symptoms to assist individuals in taking preventive actions in case of a deficiency of experts' availability. The study aspired to identify valuable patterns for COVID-19 detection from recorded data in the Kaggle dataset. Using a PART rule-based algorithm on 1,048,575 pieces of data, the model attained a 92.47% accuracy in a 10-fold cross-validation test. The authors deduced that the algorithm shows assurance, and the expert system aids disease diagnosis, offering recommendations aligned with identified symptoms. In work by (Frimpong et al. 2022), an innovative Internet of Things (IoT) system was presented for early COVID-19 detection at a low cost. The study enforced an experimental approach, designing a low-cost hardware system for students with a Wi-Fi-enabled microcontroller, a temperature detector, and a heart rate sensor. The approach efficiently detected and differentiated normal and abnormal temperature and regular and irregular heartbeat and continually depicted the student's status in a mobile application. Agreeing testing demonstrated that the forged IoT-enabled system was dependable, responsive, and cost-effective.

Like the system presented by (Frimpong et al. 2022), it is likewise essential sometimes to explore alternative approaches to help prevent the spread of the deadly virus like COVID-19. Facemasks and hand washing are among the crucial measures against the spread of viruses. While Disease Control institutions recommend wearing face masks to help slow and prevent the spread of COVID-19, experts also recommend washing reusable cloth face masks. Thus, in the same perspective, (Yadessa and Salau 2021) discussed the importance of frequent hand washing in preventing infectious diseases like COVID-19, especially in developing countries where cost-effective solutions are crucial. With the interest in incorporating embedded processors for more convenient and efficient solutions worldwide to provide effective systems for hand washing, they utilized an Arduino-based microcontroller and ultrasonic distance sensors to create a touch-free hand washing system. Their proposed design ensures that users can wash their hands without physical contact, enhancing hygiene. The authors conducted simulations using Proteus software and experiment tests to ensure particular specifications are met for effective touch-free hand washing utilization. During the COVID-19 pandemic, wearing face masks was another prevention approach to limit rapid transmission. However, in a particular case in Ethiopia, there needed to be more proof of the proportion of face mask-wearing among taxi drivers and associated factors in the country. So, as a solution, a study is presented by (Natnael et al. 2021) to reveal the proportion of facemask-wearing among taxi drivers in Dessie City and Kombolcha Town in Ethiopia. Furthermore, numerous other studies on COVID-19 detection exist in the literature (Anunay et al., 2020; Saygili, 2021; Pereira et al., 2020; Khan et al., 2020; Ahuja et al., 2021; Al-antari et al., 2021; Turkoglu, 2021; Ozcan, 2020; Ardakani et al., 2020; Göreke et al., 2021; Song et al., 2021; Wang S. et al., 2021; Zheng et al., 2020; Xu X. et al., 2020; Barstugan et al., 2020; Chen et al., 2020).

Table 1 presents a quick summary of the papers discussed in this review. Most previously reviewed literature consistently highlights the primary limitations of COVID-19 detection research studies, including the small dataset sizes and the continuous need to develop more accurate and reliable models using available methods and data. Different comparative studies used one, two, or more pre-trained models with transfer learning techniques. One thing that all of them agreed on is the need for more in-depth comparison studies that consider all possible evaluation metrics, network training hyperparameters, the model fine-tuning process, and how they might be best suited for the task at hand.

**Table 1:** Summary of the related studies.

**References**	**Study achieved**	**Used models& fine-tuning technique**	**Dataset**	**Additional preprocessing**	**Evaluation metrics**
(Jangam et al. 2022)	Stacked ensemble that detects COVID-19 using CT scans or X-rays	VGG19, Rest101, DenseNet169, and WideRestNet502 -No fine-tuning specified	Five different dataset	None	Accuracy: 98%
(Alqudah et al. 2020)	AI method that recognizes Covid-19 cases	No details provided	Details not provided	Not specified	Not provided
Apostolopoulos et al. (2020)	Transfer learning technique using X-ray scans to determine COVID-19-infected scans	No details provided	Details not provided	Not specified	Acc: 96.78%, Sens: 98.78%, Spec: 96.46%
(Ozturk et al. 2020)		Not specified	Not specified	Not specified	Acc: 98.08%, f1: 96.5%
Annavarapu et al. (2021)	Deep learning-based technique for efficient COVID-19 chest X-ray classification.	ResNet50	Not specified	Not specified	Acc: 95%
(Ozcan 2021)		ResNet50	Not specified	Not specified	Acc: 95%
(Chakraborty et al. 2022)	COVID-19 detection with performance comparison Fine-tuning Not discussed	AlexNet, VGG, ResNet, and DenseNet	10,040 samples	Image segmentation	Acc: 96.43% Sens: 93.68%
(Yadlapalli et al. 2022)	Classification of CT scans to diagnose COVID-19 patients	K-means and VGG16	COVID-19 CT scans:349 positive and 397 negative		Acc: 89 % F1: 88% AUC : 94%
(Khan et al. 2022)	Improve COVID-19 detection by Compare Three models	EfficientNetB1 fine-tuning: dropout percentage, learning rate	GitHub, Kaggle (21,165 image) Data aug. ( rotation, flipping)	Not specified	Acc: 96.13%
(Aggarwal et al. 2022)	Comparison of eight fine-tuned network	DenseNet121 and MobileNetV2	Two datasets	Data augmentation (CLAHE), image scaling and resizing	DenseNet Acc: 97% MobileEt Acc: 81%
(Rehman et al. 2021)	Detecting 15 types of chest diseases, including COVID-19, using chest X-ray modality	Two ways classification 1- Deep CNN+ soft-max 2- CNN + fully connected layer	Not specified	Not specified	Not specified
(Saeed et al. 2022)	mathematical framework called Complex Fuzzy Hypersoft (CFHS) set for diagnosing and treating medical conditions	Not specified	Not specified	Not specified	Not specified
(Allioui et al. 2022)	Long-term mask extraction for medical image segmentation	Novel multi-agent-based deep reinforcement learning (DRL) + Deep Q-Network	Not specified	CT images cases (normal, pneumonia, viral, and COVID-19)	P: 97.12%, Sens: 9.97%, Speci: 84%
(Misra et al. 2023)	Parallel ensemble transfer learning-based framework for COVID-19 (PETLFC) name for multi-class classification.	VGG16, ResNet18, and DenseNet121	Data-parallel implemented on PARAM SHAVAK HPC 21,165 chest X-ray images	Not specified	Not specified
(Ayalew et al. 2022)	DCCNet rapid COVID-19 diagnosis using X-ray images	CNN and histogram of oriented gradients (HOG) University of Gondar and online databases	Not specified	Not specified	DCCNet acc: 98% HOC acc: 98.5% Hybrid:99.67%
(Indumathi et al. 2022)	ML algorithms for forecasting current status of COVID-19	Deep C5	COVID-19 dataset from March to July 2020	Not specified	Acc: 98.06%
(Salau 2021)	Early diagnosis of COVID-19 from SARS-CoV-2	Developed discrete wavelet transform (DWT) + SVM	Not specified	Not specified	Acc: 98.2 %
(Salau 2021)	Classification of chest X-ray images as Normal or COVID-19 infected	CNN + SVM	Data from open-source	Data augmentation, image cropping, and segmentation	Acc: 99.1%
(Deriba et al. 2023)	Decision support system for Detecting coronavirus patient path based on virus' biological traits	ML models: Nave Bayes (NB), Artificial Neural Network (ANN), and Support Vector Machine (SVM)	311 patients (69% male, 31% female) collected from three Ethiopian Hospitals	Not specified	ANN R: 97 %, P: 98.3 % F1: 98.3 %
(Wubineh et al. 2023) Expert System analyzing disease diagnosing COVID-19 based on its symptoms	PART rule-based algorithm	1,048,575 pieces of data from Kaggle database	Not specified	Not specified	Acc: 92.47%

Many studies have examined respiratory diseases in general, but few have specifically focused on effectively and accurately identifying COVID-19 cases from X-ray or CT scans. Our research fills a significant knowledge gap by addressing the gaps that have not been covered in previous studies. This study proposes conducting a comprehensive analysis by training multiple models using various alternative methods due to the abundance of available data. Additionally, it employs widely recognized heuristic optimization methods, such as genetic algorithms, to identify the optimal values for hyperparameters, thereby enhancing the training process. These methodologies have demonstrated potential for improving model performance and attaining more resilient outcomes. Our study focuses on analyzing the distinct features of COVID-19 in x-ray images and utilizing these methods to enhance the development of models and training processes for accurately detecting COVID-19 cases.

The primary objective of this study is to enhance the classification accuracy and robustness of COVID-19 detection from chest X-ray images by systematically evaluating multiple deep transfer learning models under diverse training strategies. Specifically, we implement and compare three methodological paradigms: manual hyperparameter tuning, k-fold cross-validation, and genetic algorithm-based optimization, across established convolutional neural networks (CNNs) including VGG16, AlexNet, EfficientNetB1, and ResNet18. This multi-phase hybrid approach allows us to explore how different tuning methodologies impact diagnostic performance and generalizability.

In addition, we propose a novel application of the YOLOv4 object detection framework—traditionally used for real-time object localization—to the task of COVID-19 feature identification and classification in chest radiographs. Unlike existing studies that rely solely on classification networks, our work leverages YOLOv4's spatial attention capabilities to detect radiologically relevant patterns such as ground-glass opacities and bilateral consolidations. This dual-use of YOLOv4 for both region localization and diagnostic inference contributes a novel perspective to the literature by experimenting its applicability to COVID-19 X-ray classification systems.

Furthermore, we evaluate model robustness under adversarial scenarios and validate performance on an external dataset, thereby supporting the potential translational value of our proposed framework, particularly for use in resource-constrained or remote clinical settings.

## 3 Developed methodology

Most of the current research focuses mainly on enhancing convolutional neural network (CNN) models. Typically, these studies examine various crucial domains to enhance performance, efficiency, and resilience. They achieve this by employing a diverse range of models and techniques, including regularization, attention mechanisms, ensemble methods, and others. However, rather than using feature extraction or fine tuning, this study utilized the transfer learning technique by combining both strategies and implementing various training procedures with contemporary pre-trained models. One of the trainings entailed utilizing a genetic algorithm to optimize the hyperparameters of the training process. In addition, we utilized the adversarial process to assess the top model's ability to withstand potential attacks.

**Figure 1:**
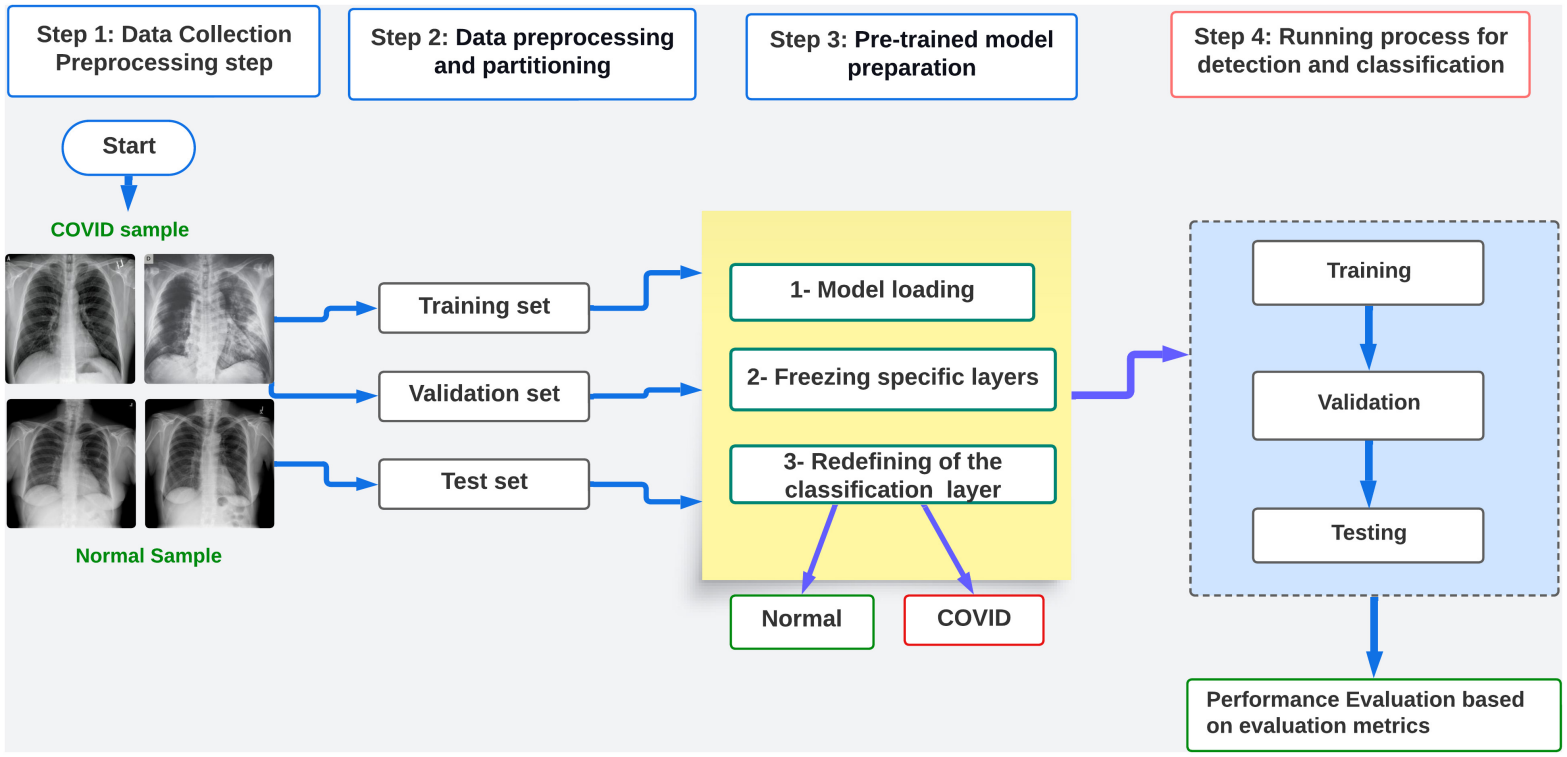
Flow chart diagram of the proposed framework.

Figure 1 illustrates the four stages of the proposed framework for detecting and classifying COVID-19 images. The first step involves gathering dependable real-world data and performing manual preprocessing tasks, such as resizing and converting images to the necessary format for the pre-trained models being utilized. In stage two, the dataset is subjected to filtering, cleaning, selection, and splitting. This process prepares the dataset for the next step by creating separate training, evaluating, and testing datasets. During the third step, the freezing process of the layers modifies and readies the models using pre-trained models, except for the classifier layer, which will only be trained.

In the fourth stage, various training procedures are implemented, including conventional training, k-fold cross-validation, and hyperparameter optimization. We conduct this optimization to evaluate the efficacy of heuristic methods on the performance of pre-trained models. We achieve this by incorporating conventional genetic algorithms (GA) to optimize hyperparameters such as learning rate, batch size, and epoch. This enables us to train the models using both training and validation data and subsequently evaluate their performance using testing data. In order to construct the suggested framework, we adhere to the subsequent procedures:

1. Dataset collection from public sources like Kaggle.
2. The preparation and processing of the dataset includes resizing, splitting, and transformation. The preprocessing is based on the model architecture.
3. Preparation of the pre-trained model: freezing and hyperparameter tuning.
4. Conducting distinct learning procedures: training and evaluating the models using the optimal hyperparameter values. Assess the performance of the model by analyzing the evaluation metrics, which include accuracy, loss, F1 score, precision, recall, AUC, and the confusion matrix.

Additionally, in order to assess the resilience of the chosen model, the most appropriate hyperparameters obtained are tested using a separate dataset. This is followed by testing the model against adversarial attacks. The study also explores the application of an object detector network, such as YOLOv4, for classifying COVID-19 x-ray images through a novel experimentation process.

### 3.1 Dataset

We have merged two openly available datasets from the Kaggle database into a unified and substantial dataset, referred to as dataset-1, for the purpose of carrying out the experimental procedure outlined in this study. The first set, the COVID-19 Radiography Dataset (Chowdhury et al., 2020; Rahman et al., 2021), consisted of 21,178 lung X-ray scans distributed across three distinct categories: COVID-19, lung opacity, and viral pneumonia. The second dataset, COVID-19 X-ray 54, comprises 2,133 images classified into three groups: COVID-19, pneumonia, and normal. We have omitted images related to other categories of classes as our research focuses solely on the COVID-19 disease. Table 2 presents the distribution of the data across all classes from both of the mentioned datasets. The distributions follow after the removal of irrelevant classes. We considered Dataset-1 to be the initial dataset that guides the entire study process. The representative images from each class are also shown in Supplementary Figure 1 (Normal) and Supplementary Figure 2 (COVID).

**Table 2:** Samples partition of the dataset.

**Dataset**	**Data labels**	**Training set**	**Validation set**	**Testing set**
	Normal	1,851	231	231
Dataset-1	COVID	1,851	231	231
	Total	3,702	462	462
	Normal	1,402	200	200
Dataset-2	COVID	1,226	200	200
	Total	2,628	400	400

Furthermore, to demonstrate the robustness and dependability of the proposed framework process, we conducted additional testing on a publicly accessible dataset (Kumar, 2022). (Shastri et al. 2022); (Kumar et al. 2022) also used this dataset in their research to analyze digital images of chest X-rays for COVID-19 disease detection. Table 2 also highlights pertinent information about this dataset, referred to as dataset 2.

### 3.2 Data preprocessing and partitioning

When preparing data for model training, several essential steps are involved. This article outlines four required stages implemented in preprocessing the obtained dataset. The initial step involved resizing all acquired images to dimensions of (224 × 224 × 1) as required for models developed in the Pytorch framework, which signifies high width and channel. The selection of 224 pixels aligns with the pre-trained networks utilized in this study, originally trained on the ImageNet dataset with a size of 224 × 224. Furthermore, considering the images are X-ray scans and have been converted to grayscale, reducing the number of channels to one is needed.

Following the image resizing process, all images within their respective class-based folders were renamed, facilitating streamlined tracking control later. In the deep learning general process, it is established that models undergo training on one set of data, validation on another, and testing on entirely new data. Therefore, data partitioning proves instrumental in ensuring the seamless progression of the deep learning model training process. Moreover, the distribution table in the preceding section revealed a certain degree of imbalance in the data. As discussed in the relevant literature section, previous studies employed data augmentation techniques to generate additional samples for classes with fewer instances to address this imbalance issue. The data augmentation process has not been applied in this study as they might result in inferior training and testing outcomes to the dataset as attempted in prior research, such as those detailed by (Ayan and Ünver 2019) and (Abdullah et al. 2020). However, some transformations were applied to the images in our study, contributing to enhanced model performance and indicating that the networks can exhibit better performance without data augmentation.

The transformation process was achieved in code using PyTorch, as it provides various predefined transformation functions tailored for each model to facilitate data preprocessing. These functions execute essential preprocessing steps on the data, encompassing tasks such as rescaling pixel values and normalization using mean and standard deviation. Both transformations were the principal ones implemented in this study.

### 3.3 Description of the proposed work

The study uses pre-trained models to process and implement deep transfer learning (DTL) information through distinct processes, as emphasized in this section. The study also investigates a novel approach that leverages the benefits of YOLOv4 using the same COVID-19 dataset. As discussed in the previous section, this study adopts an alternative method of deep learning, in line with existing research on transfer learning (Bozinovski, 2020). It involves using models that have already been trained on very large datasets to build a new architecture that can be tweaked and used to solve problems with a different dataset for a different task. It entails reusing a trained model rather than building and training a new one from scratch. The model training process becomes more adaptable and streamlined by utilizing the weights obtained from training on a large dataset, thereby reducing the amount of data needed for subsequent studies. Complex image classification tasks often employ transfer learning techniques, utilizing pre-trained models and existing labeled datasets (Sarkar, 2018; Ozturk et al., 2023; Taşyürek, 2023; Terzi et al., 2022).

Transfer learning offers numerous advantages, making it the preferred option for this study. Here are some of the reasons for this preference:

- **Data efficiency**: Trans enables deep learning models to leverage knowledge from pre-trained models on large datasets, mitigating the need for extensive labeled data in real-world scenarios, where acquiring such datasets can be costly and time-intensive. As in this study, acquiring reliable COVID-related data was more challenging than expected.
- **Fast training**: TL accelerates model training, reducing the computational expense and time required for training from scratch. This approach involves fine-tuning a model for a specific task, significantly saving computational resources.
- **Feature extraction**: By using pre-trained models as feature extractors. The lower layers of deep neural networks capture low-level features like edges, textures, or phonemes, which can be expected across different tasks. Using these features, the model can focus on learning task-specific features in the higher layers.
- **Domain and task adaptation**: TL is beneficial when the source domain (the domain on which the pre-trained model was trained) is related to the target domain (the domain of the specific task). As the domain of this study can be considered the target with limited labeled data, the knowledge from the source domain can still be beneficial for improving performance on the target task, here the classification of COVID-19 scans. The TL process implemented here enables models to adapt to new tasks without starting from scratch, making it easier to incorporate new information and update models efficiently.

### 3.4 Transfer learning strategies

**Figure 2:**
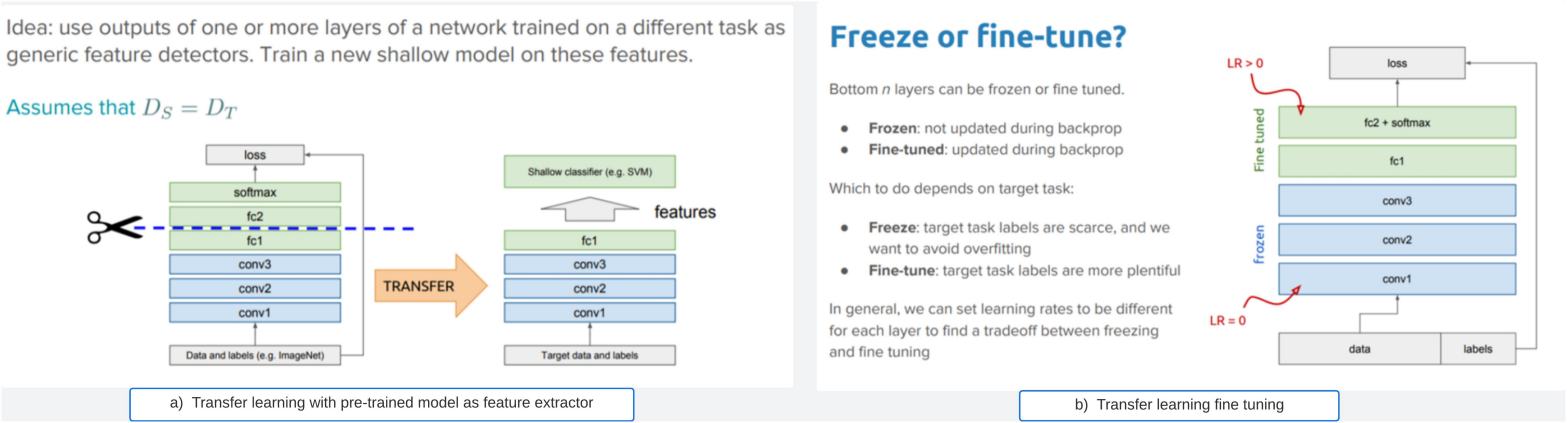
General transfer learning strategies. **(a)** Transfer learning with pre-trained model as feature extractor. **(b)** Transfer learning fine tuning.

There are two most popular strategies used when implementing deep transfer learning:

- **Feature extraction or freezing**: Unlike fine-tuning, this approach freezes the pre-trained model as a fixed feature extractor (weight does not change during training). The strategy involves conserving the pre-trained layers without the model's fully connected layers, and replacing them with a new classifier layer based on the required target task. This strategy does not update the weights during training. Figure 2a illustrates the approach. This approach is useful when the new dataset is small and similar to the original dataset used to train the pre-trained model.
- **fine-tuning**: is illustrated in Figure 2b. This strategy involves taking a pre-trained model on a source task as a starting point and adapting it to a target task. Therefore, this strategy allows for the modification or retraining of the final layers of the pre-trained model to align with the unique features of the new task. Generally, deep neural networks are known to contain highly configurable architectures with many hyper-parameters. So, when the trained model is fine-tuned, the initial layers grab generic attributes, and the later layers concentrate on specific tasks. When the new dataset closely resembles the original dataset that trained the pre-trained model, this approach becomes practical.

However, this study employed the transfer learning approach, which encompasses both freezing (for feature extraction) and fine-tuning via hyperparameters, in contrast to the majority of studies that only utilize either freezing or parameter tuning. Prior research has demonstrated that the optimal approach for refining a pre-trained model for lung X-ray scans involves keeping the first two blocks of layers fixed and subsequently training the remaining blocks (Raghu et al., 2019). Unlike the aforementioned approaches, this study chose to maintain all the pre-trained model layers fixed and frozen, except for the classifiers. We have adopted the option to extract essential features from the COVID scans and subsequently reconfigure a new classifier layer for the classification procedure. Our method utilizes all frozen layers as feature extractors and the redesigned final layer for classification, rather than just selecting two layers.

### 3.5 Pre-trained models

Numerous CNN-based architectures have proven to be effective for medical image analysis operations. Thus, in the first part of our transfer learning practice, ResNet18, VGG16, AlexNet, and EfficientNet were used as pre-trained models to build four different models. Then, in the second part, we experienced another classification model with the advantage of version 4 of the YOLOv4 model, one of the known models commonly used for object detection.

**Figure 3:**
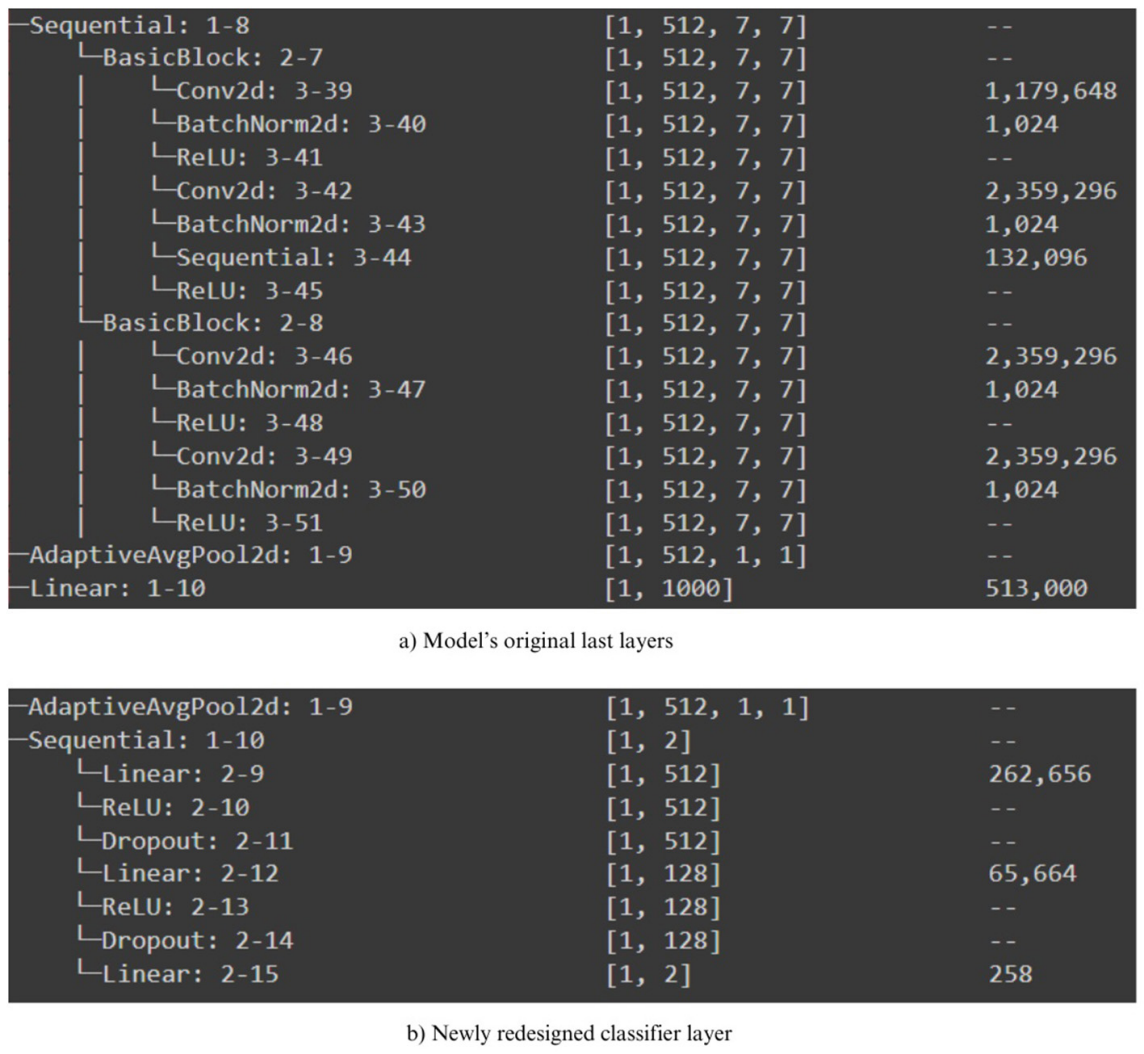
Newly redesigned classifier for pre-trained models. **(a)** Model's original las layers. **(b)** Newly redesigned classifier layer.

- **Residual neural network (ResNet18)**: This network is most prevalent in CNN architecture. Developed in 2015, it offers additional practical training with a more effortless gradient flow (He et al., 2016). As a network with **skip connections (**often referred to as "residual connections**)** that perform identity mappings merged with the layer outcomes by addition, this enables deep learning models with tens or hundreds of layers to train efficiently and approach adequate accuracy when moving deeper.
- **AlexNet**: The model was presented in 2012 as the first CNN architecture to run on GPU and participated in the ImageNet Large Scale Visual Recognition Challenge. Its architecture comprises five convolutional layers, three max-pooling layers, two normalization layers, two fully connected layers, and one softmax layer. Each convolutional layer consists of CNN filters and a nonlinear activation function, ReLU. The pooling layers are used to perform max pooling. The input size is fixed due to fully connected layers, and the model has 60 million parameters (Krizhevsky et al., 2012). AlexNet was the first deep learning model to achieve high accuracy on the ImageNet dataset.
- **Visual geometry group (VGG16)**: This CNN architecture was published in 2014 after winning the ILSVR (Imagenet) competition; it is a trained model with many hyperparameters. The model comprises many convolutional layers, 3x3 filters with a default stride of 1, followed by a 2 × 2 max pool layer. This architecture is ended with two fully connected layers. The model consists of 16 layers in total (referred to in its name), including the dropout and max-pooling layers (Simonyan and Zisserman, 2015).
- **EfficientNet**: As described by its authors in their articles (Tan and Le, 2019), the EfficientNet architecture is a deep model with a scaling method that uniformly scales all dimensions of depth/width/resolution using a compound coefficient. The scaling process of the model scales the network width, depth, and resolution based on a set of specified scaling coefficients. These parameters are uniformly scaled using a principle based on constant coefficients determined by a small grid search on the original model.

(Salau and Jain 2019) concluded in their survey paper that the distinctive characteristics that can be derived from images include homogeneity, entropy, contrast, mean, and energy. In this study, we have employed the aforementioned pre-trained models to extract potential feature extractors from the COVID-19 x-ray scans. Nevertheless, they exhibit variations from the feature extraction methods discussed in (Salau and Jain 2019). In addition, as depicted in Figure 3, we fitted all of our pre-trained architectures with an identical classifier in order to ensure an equitable assessment of performance. Subsequently, we proceeded to train the networks using the preprocessed training dataset and subsequently assessed their performance on the testing dataset. In addition, we performed supplementary testing on a distinct dataset, which was also utilized in the study proposed by (Shastri et al. 2022); (Kumar et al. 2022) to verify the performance outcomes. In addition, we enhanced this study by incorporating a testing procedure that incorporates adversarial samples, showcasing the robustness of the DTL-based optimal model against adversarial attacks. The subsequent section provides a comprehensive account of the experimental findings.

### 3.6 YOLOv4 based transfer learning

YOLOv4 is one of the most advanced models in the You Only Look Once (YOLO) family, originally developed for real-time object detection tasks. Since the release of YOLOv4 by (Alexey et al. 2020), the model has garnered attention for its enhanced detection speed and improved localization accuracy. Unlike many recent convolutional neural network (CNN) architectures that require extensive hardware resources for training (e.g., multiple GPUs and large mini-batches), YOLOv4 was designed to achieve state-of-the-art performance while remaining trainable on a single GPU with moderate batch sizes. This feature makes it particularly appealing for clinical and resource-constrained deployment scenarios.

In this study, YOLOv4 is repurposed beyond conventional object detection: we leverage its pre-trained weights and architectural strengths within a transfer learning framework for COVID-19 classification from chest X-ray images. The model's architecture—integrating CSPDarkNet53 as a backbone, along with Spatial Pyramid Pooling (SPP) and PANet path aggregation—allows it to efficiently extract multi-scale features that are crucial for identifying radiological signs such as ground-glass opacities and consolidations.

In this context, YOLOv4 is not simply used to detect real-world objects; rather, it is employed to extract meaningful spatial features that characterize COVID-19-specific manifestations in medical images. These features are then used within a hybrid pipeline to classify test samples, thereby contributing an interpretable, fast, and low-resource diagnostic alternative. This usage illustrates a novel direction in applying object detection networks for diagnostic classification in medical imaging. Thus, the reasons why YOLOv4 has been considered for experimenting in COVID-19 classification grounded in both theoretical and empirical as follows:

- Real-time efficiency: YOLOv4 is specifically designed for real-time object detection with high inference speed and low computational cost.
- Superior localization performance: Unlike earlier versions (YOLOv3) and some alternatives (e.g., SSD, Faster R-CNN), YOLOv4 integrates CSPDarkNet53 and Spatial Pyramid Pooling (SPP), which enhance multi-scale feature extraction – a critical aspect for capturing varying radiographic manifestations of COVID-19.
- Robustness to small object detection: YOLOv4's architecture demonstrates superior sensitivity to small regions of interest, which is essential in chest X-rays where lesion sizes may be subtle or localized.
- Ease of integration: YOLOv4 is compatible with transfer learning pipelines and can be easily integrated with post-classification workflows, enabling hybrid experimentation without disrupting the classification ipeline.

## 4 Experimental process and evaluation of the results

### 4.1 Experimental conditions

The experimental processes were conducted after the data were entirely preprocessed, and all the selected models were implemented, as discussed in the previous section and highlighted in the proposed framework flowchart diagram Figure 1. A well-prepared and sophisticated computing infrastructure is required to achieve the experimentations successfully. All the training processes have been performed using the pro version of the Google colab environment. The details of the computation specification are shown in Table 3. The Google Colab was preferred as it offers a more sophisticated and reliable runtime and is supported with GPU for long training processes.

**Table 3:** Computational requirements.

**Coding language**	**Python**
Google Colab Pro specifications	•GPUs: Tesla T4 (selected) •CPUs : 2 x vCPU •RAM: 32 GB

Numerous factors can dynamically influence the network's learning process, including hyperparameters such as the activation function, optimization function, batch size, learning rate, and epochs. In our study, we conducted experiments using assorted values for learning rate and considered fixed values for batch sizes and the number of epochs. The utilized values are shown in Table 4. The experiment processes were conducted in three different ways. The first was to train each model using the different selected learning rate values and compare the results. The second way is to perform the training using the K-fold scenario to ensure the performance of the models. The third was to try the training by integrating a simple genetic algorithm to experiment with different values for the three hyperparameters. Adam optimizer is employed as the optimization function for the networks with epoch and batch size set to 40 and 32, respectively, for the first and second experimental operations.

**Table 4:** Default hyperparameters used for the first training process.

**Experiment parameters**	**Values**
Learning rate	0.001, 0.0001, 0.0002
Epoch	40
Batch size	32
K-fold	5

### 4.2 K-fold-based experimentation

K-fold cross-validation is another approach operated in the literature to evaluate the performance of machine learning deep learning models. As authors in (Mahlich et al. 2025) defined, “the K-fold Cross-validation is a statistical method of evaluating and comparing learning algorithms by dividing data into two segments: one used to train the model and the other used to validate it.” The k-fold splits the dataset into k parts/fold of approximately equal size. During each epoch of the learning process, the data is distinctly split into k-parts, each for training and validation. The k-1 folds are used for training, while the remaining part is used for testing. This process is repeated k times, and then the model's performance is estimated as the average across all the test sets. This process is generally beneficial when the dataset is small, and we want to maximize the available data. The results from implementing this technique are also discussed later to demonstrate the good generalization performance of the used models.

### 4.3 Genetic algorithm-based hyperparameter tuning experimentation

A genetic algorithm (GA) is a computational optimization algorithm inspired by the principle of natural genetics based on the selection process. It is commonly operated to find the optimal or near-optimal solution to a problem by mimicking the process of evolution by iteratively evolving a population of potential solutions. GA has been an efficient technique for optimizing numerous machine-learning problems, as it has been used in the paper presented by (Li et al. 2022) to optimize convolutional neural network hyperparameters (to select trainable layers) for image classification. So, it can also be used in deep transfer learning to fine-tune a model's hyperparameters because they are not learned during the training process but are set before training begins. As mentioned in the previous descriptions, the developed method in this study utilizes deep transfer learning for COVID-19 X-ray classification, leveraging pre-trained models to expedite training while ensuring high performance even with limited labeled data. Moreover, the study also integrates a genetic algorithm to facilitate hyperparameter tuning, optimizing model parameters to enhance classification accuracy. In this study, we employed a Genetic Algorithm-based approach to optimize the identification of hyperparameter sets suitable for transfer learning models. The hyperparameters under consideration include learning rate, batch size, and the number of epochs.

In contrast to manual hyperparameter tuning, this methodology accelerates the training process and ensures efficient utilization of computational resources, thereby producing robust classification outcomes for COVID-19 detection from X-ray images. However, the standard genetic algorithm implemented during the deep transfer learning process of this study, specifically for fine-tuning hyperparameters, is summarized in the following steps::

1. **Preparation**: The dataset is prepared, and the pre-trained model is loaded.
2. **Initialization and definition of parameters' boundaries**: The hyperparameters to be optimized, including learning rate, batch size, and epochs, are defined along with their respective lower and upper boundaries.
3. **Definition of the fitness evaluation function**: For each individual, representing a combination of hyperparameters, the model is trained, and the F1 score on the validation/test set is calculated as the fitness value. The objective of the fitness function is to maximize the F1 score, thereby reflecting the model's overall performance.
4. **Initialization of the algorithm parameter values**: Key parameters such as maximum iterations, population size, and mutation and crossover probabilities are established (as required by the library package).
5. **Definition of the genetic algorithm process function**: This function is designed to execute the training process based on the hyperparameter set generated by the algorithm.
6. **Execution of the process**: The training process is carried out.
7. **Evaluation and selection of the optimal model with hyperparameter set**: The best model, along with its corresponding hyperparameter set, is evaluated and selected.
8. **Saving of the final model for future use**: The conclusive model is preserved for subsequent applications.

### 4.4 GA workflow and implementation details

The GA process was implemented using the geneticalgorithm library from Python. The library provides flexibility for managing the main flow of the GA process (selection, crossover, and mutation operations) in each iteration. It iteratively tests new hyperparameter combinations and updates the best results. The fitness function is adapted to train the model with the given hyperparameters and computes the F1 score on the validation/test set. This approach facilitates an understanding of how the generated hyperparameter sets influence the perfomance of the leaning models. This method enabled a more efficient and automated search for the best hyperparameter combinations in the search space, significantly improving the model's overall performance.

### 4.5 Detail process of genetic algorithm–based hyperparameter tuning

To systematically explore hyperparameters under a constrained compute budget, we employ a single-objective Genetic Algorithm (GA) to maximize cross–validated discrimination while regularizing training time. Let θ = (η, *b, E*) denote the learning rate, batch size, and number of training epochs, respectively. The GA searches over a bounded space (Table 5) and evaluates each candidate by *K*-fold cross-validation on the training split.

**Table 5:** GA search space and encoding.

**Hyperparameter**	**Type (encoding)**	**Range/admissible set**
Learning rate η	Real ( log_10_η∈[−5, −3] )	[10^−5^, 10^−3^]
Batch size *b*	Integer (mapped)	{16 − 32}
Epochs *E*	Integer	[10, 40]

LR is optimized in log10 space; batch size and epochs are treated as integers (rounded to the nearest admissible value).

#### 4.5.1 Search space and rationale

Learning rates are optimized on a *log scale* to cover several orders of magnitude efficiently, consistent with best practices for deep CNN fine–tuning. Batch sizes are restricted to powers of two commonly supported by commodity GPUs, and epoch bounds reflect overfitting risk observed in preliminary pilots. These ranges were chosen to (i) encompass values widely reported as effective for Models–like backbones in medical imaging, and (ii) respect our deployment–motivated compute constraints.

#### 4.5.2 GA configuration and budget

We adopt a population size of 10 and a maximum of 10 generations under our compute budget, with elitism ratio 0.1, uniform crossover probability 0.5, and mutation probability 0.15 (Gaussian perturbation in log–LR; neighbor moves for integers). Premature convergence is mitigated via (i) diversity preservation by re-seeding up to 10% of the population every 5 generations, and (ii) a stagnation stop if the best fitness improves by < 10^−3^ over generations. To ensure reproducibility, we fix the GA and data-split seeds and report the selected parameter set.

### 4.6 Performance metrics

The experimented models for the presented framework are evaluated based on the commonly used metrics in the literature for assessing classification performance. Then, a comparison of results between the proposed models and recent results from the literature is discussed in Section.

In classification problems, the following terms are used to define the metrics:

- **True positive (TP):** The number of instances correctly predicted as belonging to the positive class.
- **False positive (FP):** The number of instances incorrectly predicted as belonging to the positive class (actually negative).
- **True negative (TN):** The number of instances correctly predicted as belonging to the negative class.
- **False negative (FN):** The number of instances incorrectly predicted as belonging to the negative class (actually positive).

These definitions are essential for understanding the evaluation metrics presented in Table 6.

**Table 6:** Performance evaluation metrics.

**Metrics**	**Equation formula**
Precision (P)	TP/(TP + FP)
Recall (R)	TP/(TP +FN)
Accuracy	(TP + TN)/(TP + TN + FP + FN)
F1-score	2 [P-R/(P+R)]

Here, TP, True Positive; FP, False Positive; TN, True Negative; FN, False Negative.

## 5 Results evaluation and discussion

This section highlights the results obtained from all the experimental processes carried out as explained previously and also presents an evaluation of the performance comparisons of the models. This performance comparison is based on the evaluation metrics [accuracy, precision, recall, F1-score score, and the Area Under the Curve (AUC)] acquired from the testing process. In addition to these metrics, the models are also relatively compared alternatively according to the results achieved during training, validation, and testing by considering their accuracy and loss. Generally, the best model is the model having the highest accuracy, F1-score, and AUC with the lowest loss in testing (Odeh et al., 2022), proving that the model has no underfitting or overfitting issue for the intended task, which is the classification in this study. Furthermore, an additional comparison is also discussed to compare the models based on the execution time spent by each model for the evaluation steps to achieve the best accuracy. This section is subdivided into three subsections. The first discusses the results of the applied transfer learning for each model trained with three different sets of hyperparameters. The second subsection discusses the results achieved by the same models based on the k-fold training process to confirm the results of the best model pointed out in the later subsection. The thirst concerns genetic-based computation.

### 5.1 Transfer learning-based results

As mentioned in the experimental section, a set of different hyperparameter sets has been manually chosen after conducting several tries. Eventually, three sets have been selected as near-to-optimal values to help models achieve optimal accuracy with low loss. These hyperparameters are presented in the “hyperparameters” column of Table 7 for each model, such as learning rate(Lr), batch size (b.size), and epoch (ep).

**Table 7:** Models' performance results from transfer learning.

**Model**	**Hyperparam. Lr/b.size/ep**	**Training acc**	**Valid acc**	**Test acc**	**F1 score**	**P**	**R**	**AUC**	**Exec time(s)**
(a)	0.001/32/40	99.20	97.4	99.55	99.35	99.13	99.57	99.57	3.561
ResNet18	0.0001/32/40	99.20	98.90	99.55	99.35	99.13	99.57	99.57	3.224
	0.0002/32/40	99.5	98.80	99.78	99.67	99.56	99.78	99.80	3.205
(b)	0.001/32/40	98.8	98.3	98.70	98.71	98.73	98.70	99.71	2.636
AlexNet	0.0001/32/40	99.3	98.20	98.92	98.92	98.93	98.92	98.93	2.794
	0.0002/32/40	99.3	98.2	98.70	98.70	99.13	98.26	98.70	2.820
(c)	0.001/32/40	99.3	98.8	98.70	98.06	97.40	98.06	98.06	5.065
VGG16	0.0001/32/40	99.5	97.8	97.80	98.00	97.80	97.80	97.87	4.817
	0.0002/32/40	99.4	97.8	98.92	98.91	98.93	98.92	98.93	4.597
(d)	0.001/32/40	99.3	98.8	98.7	97.19	97.26	97.19	97.25	2.636
EficientNet	0.0001/32/40	99.5	97.8	97.40	97.37	97.42	97.40	97.40 2.794
B1	0.0002/32/40	99.4	97.8	98.48	98.46	98.40	98.92	98.50	2.820

Lr, learning rate; b.size, batch size; ep, epoch; acc, accuracy.

**Figure 4:**
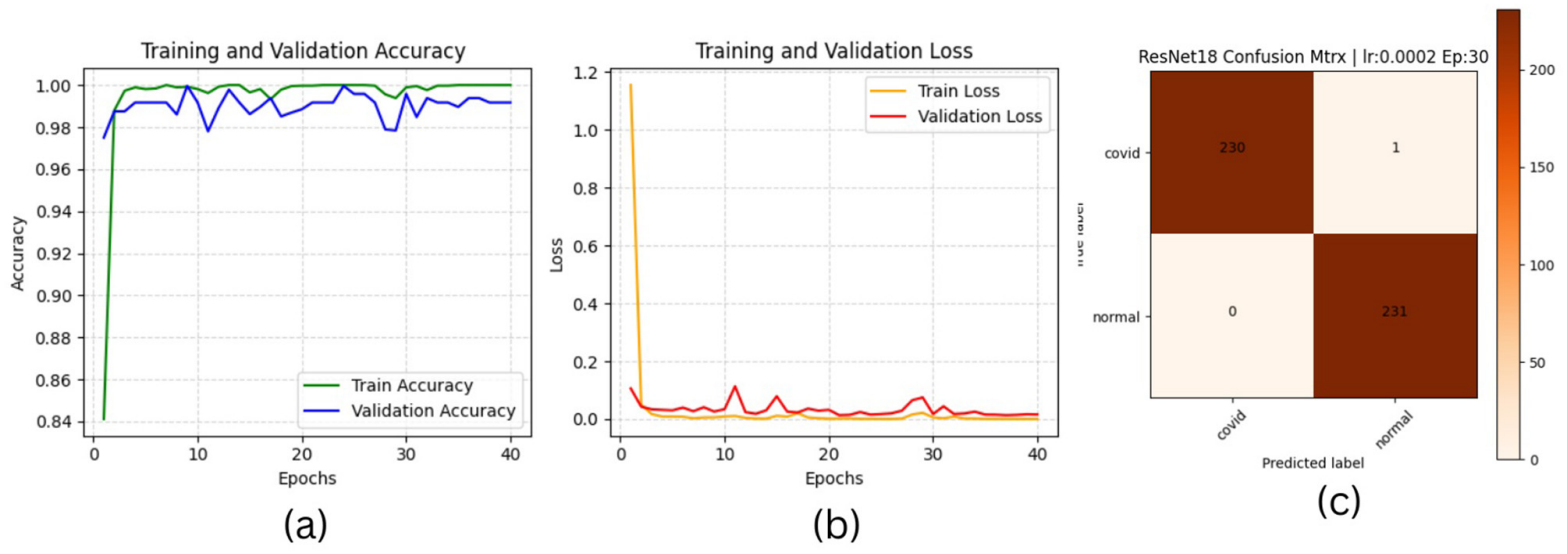
ResNet18 plotting results **(a)** accuracies, **(b)** losses, and **(c)** confusion matrix of the testing.

**Figure 5:**
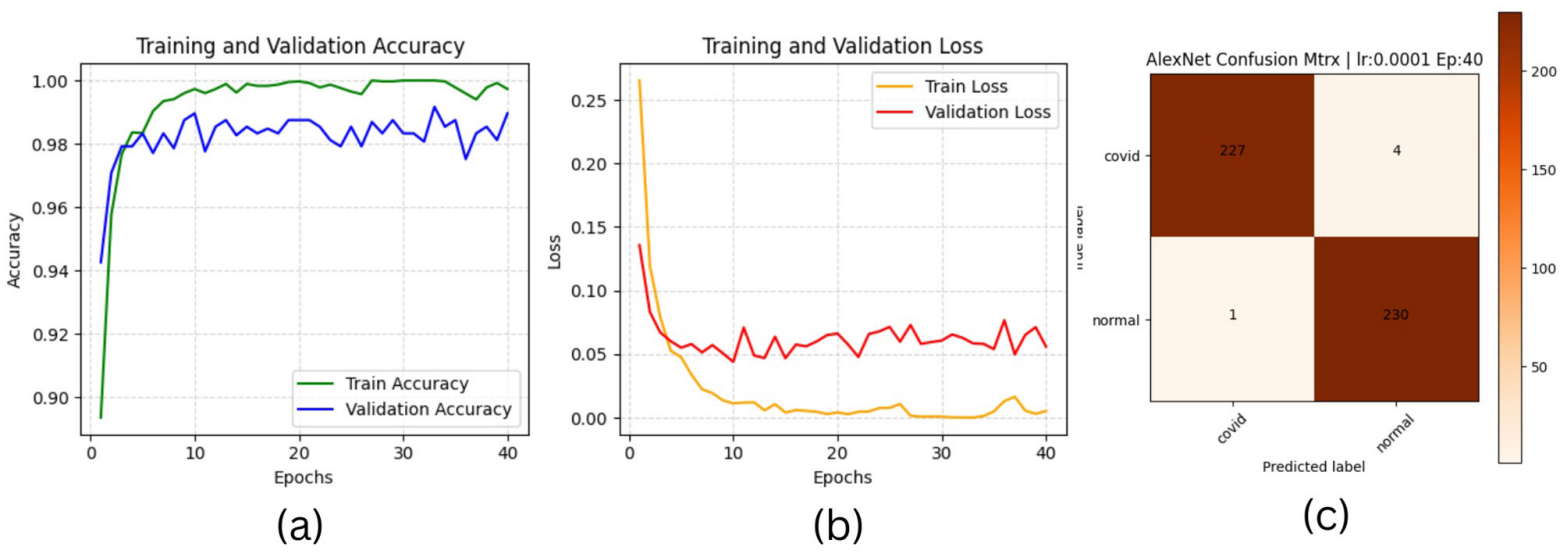
AlexNet plotting results **(a)** accuracies, **(b)** losses, and **(c)** confusion matrix of the testing.

**Figure 6:**
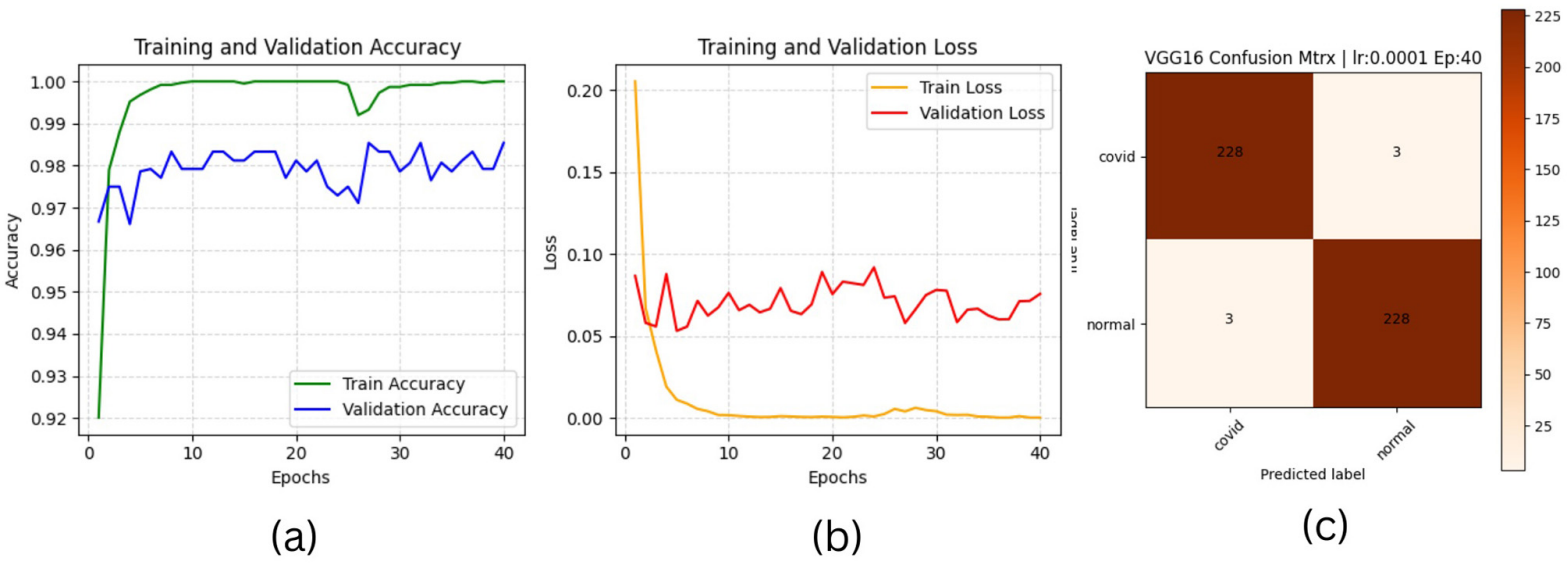
VGG16 plotting results **(a)** accuracies, **(b)** losses, and **(c)** confusion matrix of the testing.

**Figure 7:**
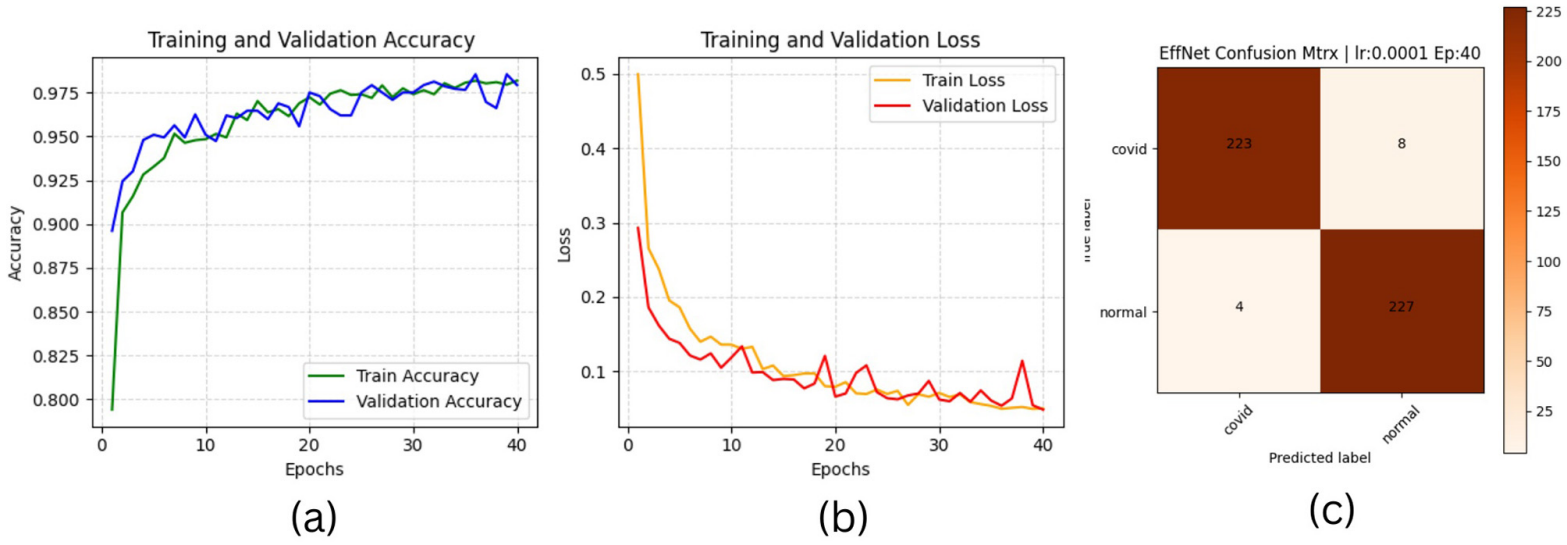
EfficientNetB1 plotting results **(a)** accuracies **(b)** losses and **(c)** confusion matrix of the testing.

The results of “RestNet18” and AlexNet models are provided in Tables 7a, b respectively, which shows they achieved better performance with the same hyperparameters set. The table highlights each hyperparameter set's training, validation, and testing outcomes. These results demonstrate that decreasing the learning rate with fixed epochs and batch size can yield better results, as it can be viewed with a slight increase in training, validation and testing accuracies and a decrease in losses. The highest F1-score, precision, recall and average AUC have been achieved with the (lr:0.0001, bs:32, ep:40) parameter set for each of both models. Figures 4a–c, 5a–c shows the graphical evolution of the training and validation accuracies and losses for both models and the confusion matrix yielded from the testing process. On the other hand, sharing similar hyperparameters set (lr:0.0001, bs:32, ep:40) with their better results, the experimental outcomes for VGG16 and EfficientNetB1 as presented in Tables 7c, d, their accuracies, losses and confusion matrices are shown in Figures 6, 7 restively. Looking to result in Table 7c, the training and validation accuracies and losses of the VGG16 do not present a big difference for both sets (lr:0.0001, bs:32, ep:40) and (lr:0.0002, bs:32, ep:40), however, exhibited a remarkable difference for testing and remaining metrics (F1-score, precision, recall, and AUC) with satisfactory results with (lr:0.0002, bs:32, ep:40) set.

Furthermore, from the point of view concerning the execution time expended by each model to complete the testing process, the analysis of these times in the corresponding tables confirms it is slightly decreasing relative to the decrease of the learning rate, which also proves that a pre-trained model can be trained with a low rate for better performance in short time. More detailed information, including the results of the training process, accuracies and loesses are presented in Table 8.

**Table 8:** Models' performance results from transfer learning with details.

**Model**	**Hyperparam. Lr/b.size/ep**	**Training acc**	**Training loss**	**Valid acc**	**Valid loss**	**Test acc**	**Test loss**	**F1-score**	**P**	**R**	**AUC**	**Exec time(s)**
(a)	0.001/32/40	99.20	0.026	97.4	0.09	99.55	0.030	99.35	99.13	99.57	99.57	3.561
ResNet18	0.0001/32/40	99.20	0.061	98.90	0.04	99.55	0.006	99.35	99.13	99.57	99.57	3.224
	0.0002/32/40	99.5	0.035	98.80	0.035	99.78	0.007	99.67	99.56	99.78	99.80	3.205
(b)	0.001/32/40	98.8	0.034	98.3	0.071	98.70	0.065	98.71	98.73	98.70	99.71	2.636
AlexNet	0.0001/32/40	99.3	0.021	98.20	0.064	98.92	0.047	98.92	98.93	98.92	98.93	2.794
	0.0002/32/40	99.3	0.027	98.2	0.072	98.70	0.045	98.70	99.13	98.26	98.70	2.820
(c)	0.001/32/40	99.3	0.022	98.8	0.079	98.70	0.064	98.06	97.40	98.06	98.06	5.065
VGG16	0.0001/32/40	99.5	0.015	97.8	0.08	97.80	0.057	98.00	97.80	97.80	97.87	4.817
	0.0002/32/40	99.4	0.015	97.8	0.091	98.92	0.045	98.91	98.93	98.92	98.93	4.597
(d)	0.001/32/40	99.3	0.022	98.8	0.079	98.7	0.064	97.19	97.26	97.19	97.25	2.636
EficientNet	0.0001/32/40	99.5	0.015	97.8	0.08	97.40	0.057	97.37	97.42	97.40	97.40	2.794
B1	0.0002/32/40	99.4	0.015	97.8	0.091	98.48	0.045	98.46	98.40	98.92	98.50	2.820

### 5.2 K-fold based models performance and confusion matrix

As the second learning process achieved in this study, the k-fold process has been performed to ensure the models' generalization. The previous results have been obtained based on the manual traditional data splitting technique (train, validation, and test dataset) to train all the models. However, to perform the k-fold operation, the train and validation datasets (from the previous process) are joined together and passed to the k-fold algorithm, which splits the provided dataset in train and valid at each fold to perform the training and validation. At the same time, the testing is achieved using the test dataset. The model performance results at each fold are presented in Tables 9a–d, respectively. The last row of each model's row highlights the average results (from the overall folds) for each evaluation metric.

**Table 9:** K-5-based results of models.

**Models**	**K-fold steps**	**Training acc**	**Training loss**	**Valid acc**	**Valid loss**	**Test acc**	**Test loss**	**F1-score**	**P**	**R**	**AUC**
(a) ResNet18	K-1	98.86	0.077	98.35	0.057	99.57	0.028	99.57	99.57	99.57	99.7
	K-2	99.8	0.008	99.62	0.012	99.35	0.038	99.35	99.36	99.35	99.2
	K-3	99.75	0.009	99.96	0.002	99.78	0.007	99.78	99.78	99.78	99.78
	K-4	99.88	0.005	99.92	0.002	99.57	0.011	99.57	99.57	99.57	99.57
	K-5	99.89	0.004	99.95	0.001	99.57	0.010	99.57	99.57	99.57	99.57
	Averages	99.64	0.020	99.56	0.014	99.57	0.019	99.57	99.57	99.57	99.57
(a) AlexNet	K-1	99.22	0.021	96.72	0.058	98.82	0.060	98.92	98.94	98.92	98.7
	K-2	99.86	0.005	99.82	0.005	98.48	0.097	98.48	98.49	98.48	97.8
	K-3	99.92	0.003	99.97	0.001	98.78	0.060	99.78	98.78	98.78	99.78
	K-4	99.91	0.003	99.92	0.002	99.13	0.044	99.57	99.14	99.13	99.10
	K-5	99.95	0.002	99.95	0.001	99.35	0.058	99.57	99.36	99.35	99.30
	Average	99.77	0.006	99.29	0.033	99.35	0.019	99.57	99.35	99.35	99.35
(a) VGG16	K-1	99.47	0.014	97.61	0.018	99.35	0.039	99.35	99.36	99.35	98.7
	K-2	99.9	0.004	99.9	0.083	98.27	0.118	98.27	98.28	98.27	97.8
	K-3	99.93	0.002	99.93	0.007	98.48	0.053	98.48	98.53	98.53	98.48
	K-4	99.95	0.002	99.99	0.004	98.7	0.047	98.7	98.7	98.7	98.9
	K-5	99.96	0.001	99.8	0.001	99.13	0.088	99.14	99.14	99.1	99.02
	Averages	99.84	0.024	99.97	0.027	99.13	0.069	98.79	99.14	99.13	99.13
(a) EfficientNetB1	K-1	95.52	0.019	93.66	0.163	98.05	0.07	96.97	96.98	96.97	98.7
	K-2	98.63	0.039	98.09	0.053	96.97	0.088	96.97	97.1	96.97	97.8
	K-3	98.55	0.04	99.88	0.006	98.27	0.048	98.27	98.27	98.27	99.78
	K-4	98.9	0.031	99.93	0.004	98.7	0.037	98.7	98.72	98.7	98.7
	K-5	99.14	0.025	99.99	0.001	98.27	0.065	98.27	98.28	98.27	98.4
	Averages	98.15	0.006	98.31	0.045	98.27	0.061	97.84	98.28	98.27	98.27

The k-fold (5-fold) results of the RestNet18 and AlexNet models are presented in Table 9a. Their average results for each metric are almost the same as pointed out in the previous process. The ResNet averaged 99.57 for test accuracy and 99.57 for F1-score, precision, recall and AUC, respectively. However, AlexNet registered an improvement by averaging an accuracy of 99.35 from 98.92 in the previous process. It averaged 99.57 for F1-score and 99.35 for precision and recall compared to 98.92 for these metrics results scored previously. This improvement demonstrates the K-fold process's efficiency in improving the models' performance.

**Figure 8:**
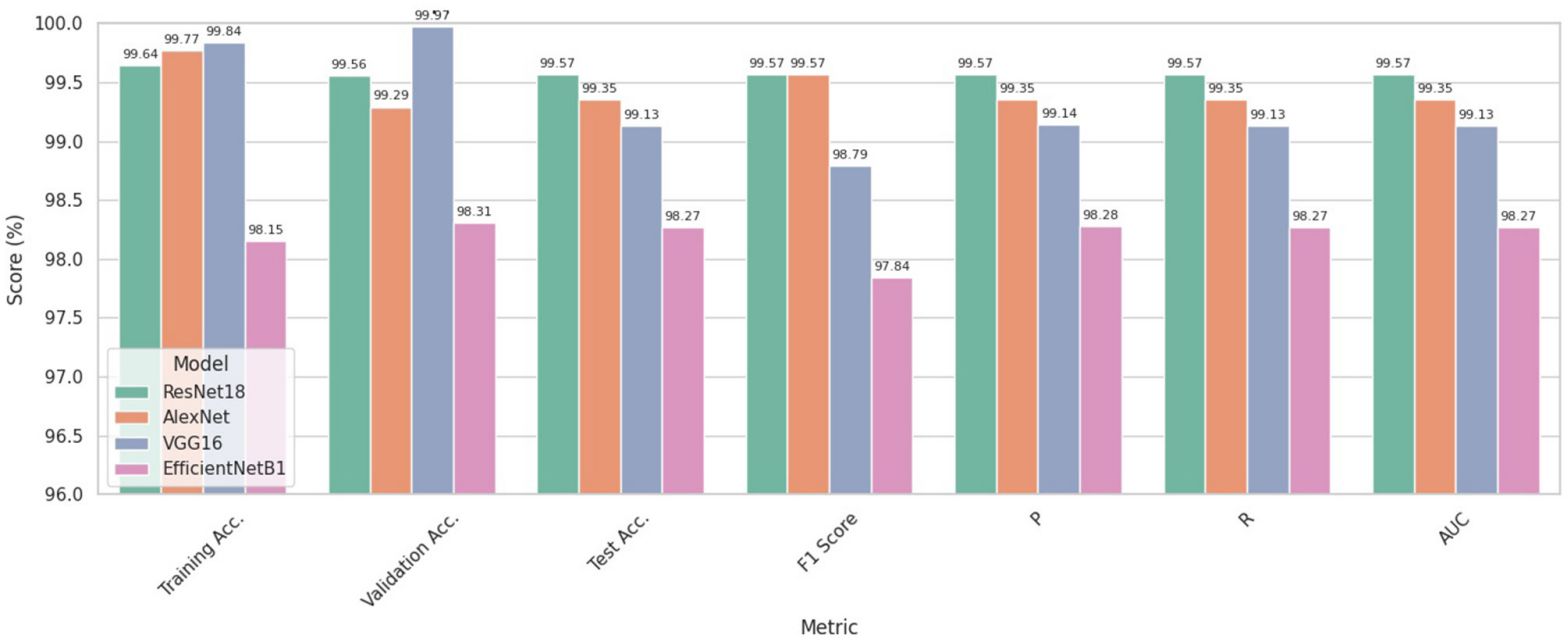
Models' Performances Based on the k1-5 average on each metric.

Figure 8 illustrates the average performance outcomes for all models, assessed using various metrics across all five folds.

On the other hand, Tables 9c, d highlight the 5-fold result for VGG16 and EfficientNetb1 models as both have been trained with the same parameter sets (0.0002, 32,40) for five-folds, considering that this hyperparameter set provided the best results for both models in the previous process. These results again confirm the slight improvement of both models. The VGG model improved from 98.92 to 99.13 for test average accuracy and decreased in testing loss from 0.069 to 0.045. Furthermore, it has maintained the same view improvement for F1-score, precision and recall. However, unlike other models, the efficientNet1 needed to register sufficient improvement, probably due to the structure of the model or the use of hyperparameters that did not impact the model's performance. More detailed information, including the results of the training process, accuracies and loesses are presented in Table 8.

### 5.3 GA-based model performance results

**Figure 9:**
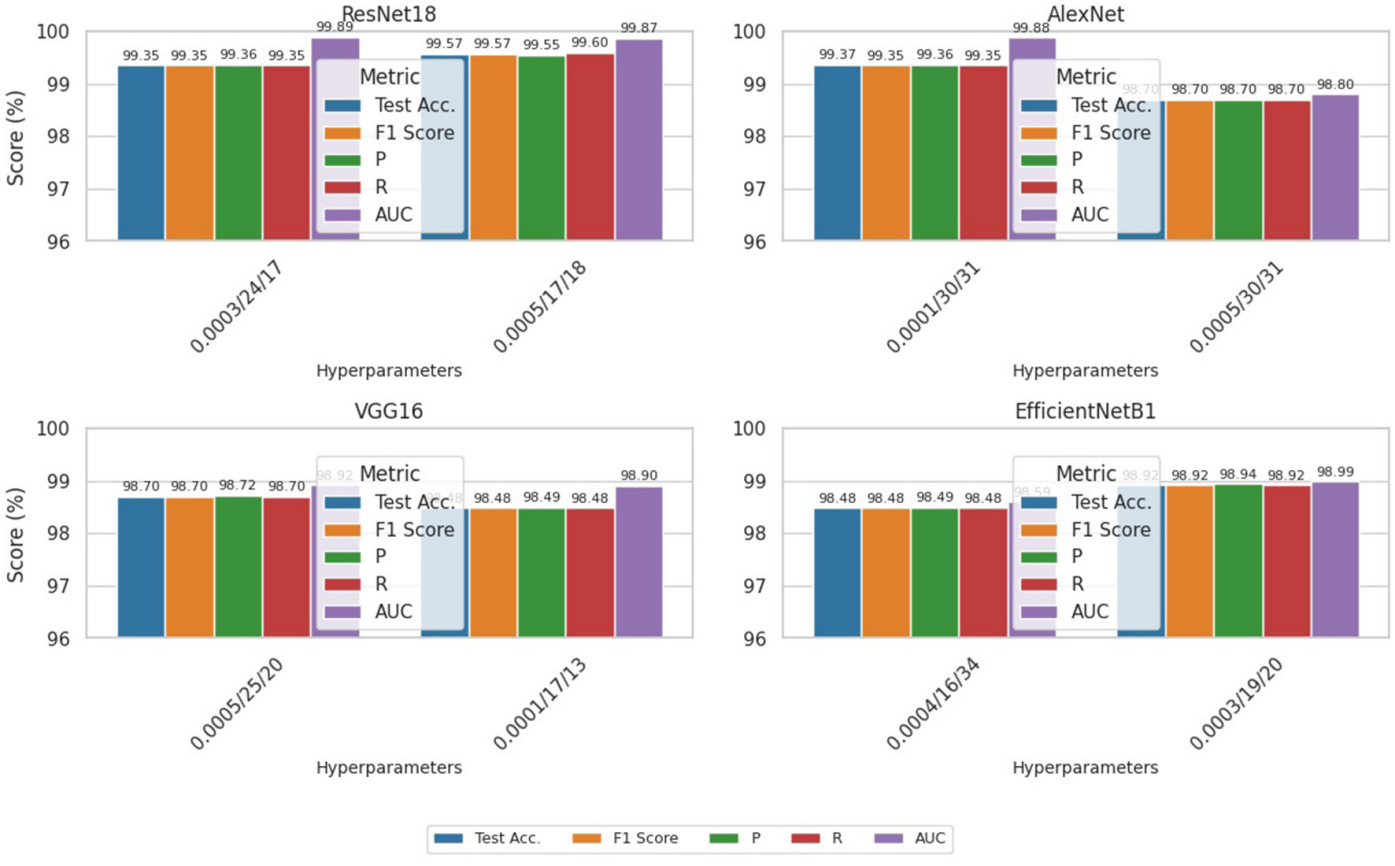
Comparative performance per models (per GA-based hyperparameter set).

As the third experimental process explored in this study, genetic algorithms have been used to determine different hyperparameters for training and testing the model's performance. This process was carried out to try automatic hyperparamet settings instead of manual settings, as achieved in the first process. The performance evaluation results based on the evaluation metrics are highlighted in Table 10. This Table presents the training, validation and testing results (accuracy and loss), followed by the F1-score, precision, recall and AUC for each model according to each defined value for hyperparameters such as learning rate, batch size and epoch. The integrated GA running process has been performed for ten iterations, and only the top parameter sets that have provided better results for the models are shown in Table 10. However, the analysis of these results also reveals that ResNet18 has outperformed the other models with different sets of hyperparameters (lr:0.0003,bs:24, ep:17) and (lr:0.0005,bs:17, ep:18) where the model reaches almost 100% accuracy for training, 99.57% for validation and testing and F1-score of 99.57. The AlextNet and VGG16 models also have training accuracies of more than 99.5%, while the validation, testing and F1-score is near 99%. These experience outcomes demonstrate that pre-trained models could be trained and validated with low learning rates and epochs to achieve remarkable results. The graphical presentation of this comparative results is shown in Figure 9. The performance of all the models evaluated above has been compared in detail using metrics such as accuracy, precision, recall, and F1 score. The results of these comparisons are presented in Table 11.

**Table 10:** GA-based models' performance results.

**Model**	**Hyperparam. Lr/b.size/ep**	**Training acc**	**Training loss**	**Valid acc**	**Valid loss**	**Test acc**	**Test loss**	**F1-score**	**P**	**R**	**AUC**

(a)	0.0003/24/17	100	0.0001	99.35	0.037	99.35	0.038	99.35	99.36	99.35	99.89
ResNet18	0.0005/17/18	99.93	0.0019	99.57	0.0622	99.57	0.0722	99.57	99.55	99.60	99.87
(b)	0.0001/30/31	99.95	0.0013	99.35	0.0888	99.37	0.0768	99.35	99.36	99.35,	99.88
AlexNet	0.0008/18/34	99.62	0.0118	98.92	0.0765	98.92	0.0664	98.92	98.92	98.92	98.89
	0.0005/30/31	99.89	0.0044	98.7	0.1404	98.7	0.1304	98.7	98.7	98.7	98.80
(c)	0.0005/25/20	99.89	0.0035	98.7	0.1073	98.7	0.120	98.7	98.72	98.7	98.92
VGG16	0.0001/17/13	99.04	0.0282	98.48	0.0671	98.48	0.0574	98.48	98.49	98.48	98.90
	0.0002/18/28	100	0.0002	98.92	0.0811	98.92	0.0714	98.92	98.92	98.92	98.99
(d)	0.0005/18/20	98.92	0.0312	98.7	0.055	98.7	0.036	98.7	98.7	98.7	98.79
EficientNetB1	0.0004/16/34	98.4	0.0452	98.48	0.06	98.48	0.0590	98.48	98.49	98.48	98.59
	0.0003/19/20	99.58	0.0131	98.92	0.0624	98.92	0.0432	98.92	98.94	98.92	98.99

Lr, learning rate; b.size, batch size; ep, epoch; acc, accuracy.

**Table 11:** Performance summary of all models under different experimental strategies.

**Model**	**Experiment**	**Accuracy (%)**	**F1-score (%)**	**Precision (%)**	**Recall (%)**	**AUC (%)**
EfficientNetB1	Manual hyperparameter	98.48	98.46	98.40	98.92	98.50
VGG16	Manual hyperparameter	97.80	98.00	97.80	97.80	97.87
AlexNet	Manual hyperparameter	98.92	98.92	98.93	98.92	98.93
ResNet18	Manual hyperparameter	99.78	99.67	99.56	99.78	99.80
EfficientNetB1	K-fold average	99.13	98.79	99.14	99.13	99.13
VGG16	K-fold average	99.35	99.57	99.35	99.35	99.35
AlexNet	K-fold average	99.57	99.57	99.57	99.57	99.57
ResNet18	K-fold average	98.27	97.84	98.28	98.27	98.27
EfficientNetB1	GA-based Hyperpar.(0.0003/19/20)	98.92	98.92	98.92	98.92	98.89
VGG16	GA-based Hyperpar.(0.0002/18/28)	98.92	98.92	98.92	98.92	98.99
AlexNet	GA-based Hyperpar.(0.0001/30/31)	99.37	99.35	99.36	99.35	99.88
**ResNet18**	**GA-based Hyperpar.(0.0005/17/18)**	**99.57**	**99.44**	**99.55**	**99.60**	**99.87**
YOLOv4	Normal TL process	90.58	94.5	90	0.928	94.60

Bold values indicate the best-performing results across all metrics.

### 5.4 Robustness of experimented models gainst adversarial attack

**Figure 10:**
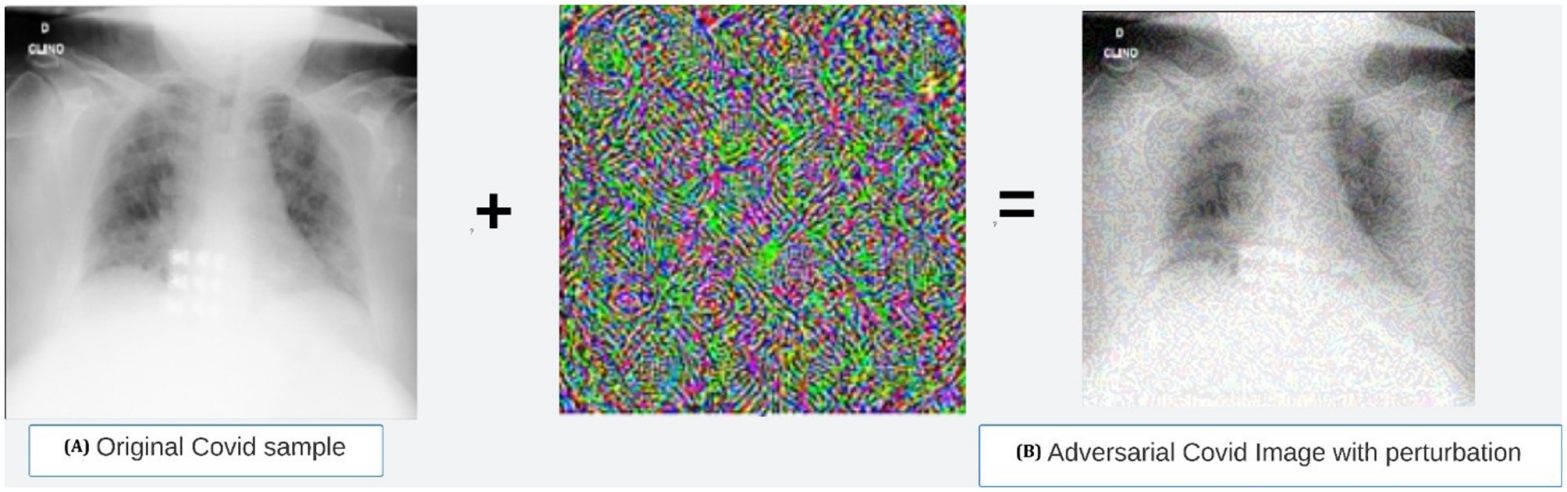
Adversarial COVID-19 sample generated using FGSM method. **(a)** Original Covid sample. **(b)** Adversarial COVID image with perturbation.

This section focuses on the developed vulnerability to adversarial attacks, which can cause models to misclassify an adversarial sample with high confidence. Researchers have found that making small changes to normal samples can create adversarial examples that trick DNN-based models into giving wrong predictions. Despite numerous studies discussing possible adversarial attacks on classification models, none have discussed and tested these attacks on classifier COVID-19 models with adversarial examples. Researchers have employed various techniques, including the dense adversarial method, random label assignment, and metaheuristics, to generate adversarial samples. Khan et al. created a novel algorithm based on metaheuristics, drawing inspiration from the behavior of a beetle, which can deceive CNNs in classification tasks by disrupting a single pixel in an input image. Szegedy et al. developed adversarial training, which is known to be the better way for DNN models to be protected against perturbation. Thus, to ensure and enhance the robustness of the ResNet18-based model, we've implemented adversarial training and testing. Initially conceived for the classification task, the Fast Gradient Sign Method (FGSM) (Goodfellow et al., 2014) and Projected Gradient Descent (PGD) (Madry et al., 2017) are two well-known and popular adversarial sample generator methods. Thus, the paper adopted FGSM to generate adversarial images to retrain the models. Equation 1 expresses the FGSM-based attack's application, which generates perturbed images by adjusting (perturbing) the original input sample image x by the amount of epsilon along the gradient direction. The process of generating adversarial images based on the FGSM on the dataset used in this study is shown in Figure 10. The used epsilon value is 0.2 to create the perturbation.


(1) $$\begin{array}{l} x_{adv}=x+\epsilon .sign\left({▽}_{x}\ell \left(h\left(X\right),y\right)\right) \end{array}$$

Table 12 shows the results of the model accuracies after adding the generated adversarial examples to the initial dataset for retraining, thus to prove the robustness of the best model.

**Table 12:** ResNet18 model performance on both datasets and with adversarial samples included.

**Training process**	**Training acc**	**Training loss**	**Valid acc**	**Valid loss**	**Test acc**	**Test loss**	**F1-score**	**P**	**R**	**AUC**
(a) Dataset-2 results	99.93	0.0019	99.57	0.0622	99.57	0.0722	99.57	99.57	99.57	99.87
(b) Dataset-1 results	99.50	0.0911	99.30	0.2303	99.35	0.063	99.36	99.37	99.36	99.89
(c) Dataset-2 results	99.20	0.2131	98.90	0.1703	99.20	0.078	99.26	99.37	99.36	99.88

## 6 Discussion, comparison, clinical application, and limitation

### 6.1 Discussion and comparison

This section provides a comprehensive analysis and comparison of the current study with related state-of-the-art research.

- Based on the investigation of our study results, we found that models based on ResNet18, AlexNet, and VGG16 exhibited the highest test accuracies, exceeding 99%, with scores of 99.57%, 99.35%, and 99.13%, respectively. In numerous studies, these models have demonstrated superior performance in the classification of COVID-19 and normal classes. For example, works such as (Jangam et al. 2022), (Aggarwal et al. 2022), and (Misra et al. 2023), which also conducted comparative analyses, illustrate the superior efficacy of these models. The results of the remaining evaluation metrics, including F1-score, precision, and recall, as presented in various tables in the preceding sections, corroborate these findings.
- Table 13 illustrates the superior performance of the optimal model presented in this research relative to prior studies. This comparative analysis encompasses a varied collection of datasets and neural network architectures, alongside the results attained by the most effective models from each referenced study. By utilizing appropriate evaluation metrics, we evaluated the performance of each of our models and established that the majority exhibited outstanding results.
- The EfficientNetB1 model exhibited the lowest performance, achieving an accuracy of 98.27% during testing. Nonetheless, this accuracy remains higher than the 96.13% reported by (Khan et al. 2022) in their investigation, which compared the EfficientNetB1 pre-trained model against two other models.
- Furthermore, the VGG16 model surpassed the performance of the VGG16 model proposed by (Yadlapalli et al. 2022), achieving an accuracy score of 89%. Additionally, it outperformed the model presented by (Ozturk et al. 2023), which recorded an accuracy score of 98.8%.
- (Aggarwal et al. 2022) and (Misra et al. 2023) have conducted comprehensive research that encompasses the study by (Odeh et al. 2022) and additional comparative analyses, such as that by (Yang et al. 2021), demonstrating the efficacy of DenseNet121 in the classification of X-ray images. In a distinct investigation, (Hasan et al. 2021) utilized DenseNet to accurately predict COVID-19 in CT images, achieving an accuracy rate of 92%.
- Moreover, a comparative study conducted by (Shazia et al. 2021) assessed DenseNet121 in relation to several other models, including VGG16, VGG19, Inception-ResNet-V2, and InceptionV3. The findings revealed that DenseNet121 surpassed the performance of the other architectures with a success rate of 99.48%.
- Furthermore, as highlighted in the comparative table, numerous studies, including those conducted by (Odeh et al. 2022), (Chakraborty et al. 2022), (Aggarwal et al. 2022), and (Ozcan 2021), have demonstrated the efficiency of models such as InceptionV3, VGG16, and ResNet50, indicating their suitability for medical image classification. The work of (Mukesh et al. 2023), which compared and analyzed the crack detection performance of VGG16, InceptionV3, and ResNet50, reached a similar conclusion. In their study, (Kamal and Ez-Zahraouy 2023) utilized VGG16, VGG19, and ResNet50 architectures to classify medical images into distinct categories. Additionally, (Srinivas et al. 2024) presented a model that integrated InceptionV3 and VGG16, achieving the highest accuracy of 98% when compared to other deep learning models such as ResNet50, DenseNet121, and MobileNet for COVID-19 prediction.
- However, alternative models such as ResNet18, despite not being frequently employed in other studies, have proven to be the most effective choice in this research, yielding satisfactory outcomes. It achieved a test accuracy of 99.57% for F1-score, precision, recall, and average AUC. These metric-based results surpass those reported in recent studies, including (Odeh et al. 2022) and (Aggarwal et al. 2022). Various recent investigations, including those focusing on COVID-19 detection by (Al-Falluji et al. 2021), (Khanal et al. 2024), (Kakarwal and Paithane 2022), and (Liu et al. 2021), have demonstrated the efficacy of ResNet18 for medical imaging tasks.
- This study underscores the importance of experimenting with diverse use cases and selecting appropriate hyperparameters to elucidate the quality of model performance. The research conducted by (Iqbal et al. 2022) and (Cejudo Grano de Oro et al. 2022) further emphasizes the considerable influence of manual hyperparameter selection on the performance of deep learning models. While this study has successfully integrated the genetic algorithm (GA) process for optimal hyperparameter selection, thereby reducing the time-consuming nature of manual hyperparameter determination, it has achieved comparable results.
- Additionally, as previously indicated, the EfficientNetB1 network produced inferior results compared to its counterparts, potentially due to its relatively shallow architecture. InceptionV3 (48 layers), which is comparable to ResNet50 (50 layers) and DenseNet121 (121 layers), exemplifies a network with a greater number of layers achieving superior results. Nonetheless, ResNet18 (18 layers) performs comparably to ResNet50, and our research indicates that networks with fewer layers can also achieve satisfactory performance when appropriately trained with the correct data and optimized hyperparameters. This finding aligns with the results reported by (Farag et al. 2021).
- Furthermore, these observations validate the practicality of the residual block technique over the inception module technique introduced in Inception V3. Poorly selected hyperparameters can occasionally result in overparameterization issues during training, leading to diminished performance in networks characterized by a greater number of layers.
- Furthermore, the YOLOv4-based model employed in this study achieved an accuracy of 86%, which is relatively low in comparison to other reported results. This discrepancy may be attributed to the learning paradigm utilized. Typically, the learning process incorporates either fully supervised or weakly supervised learning, as examined in previous research (Chen et al., 2022; Wang H. et al., 2021; Ren and Cai, 2023). In instances where the dataset is sufficiently large, fully labeled, and free of noise, fully supervised learning is preferred. Conversely, weakly supervised learning is more advantageous when dealing with datasets that contain noise, as was the case in our training process with YOLOv4. During object detection, the YOLO algorithm endeavors to learn not only the object itself but also its surrounding context. In this study, the implementation of weakly supervised learning resulted in the entire COVID-19 scan being labeled as a single object. Consequently, the performance of the YOLOv4 model was inferior to that of other models, as its approach aimed to identify all potential objects within the images, leading to the misclassification of COVID scans. Rather than utilizing the entire image for this process, fully supervised learning would represent a more effective strategy. This would involve the precise identification and labeling of the COVID-19 region within the image, followed by retraining the model to achieve superior performance compared to other models. Future studies will explore this approach to enhance model efficacy.

**Table 13:** Comparison with previous state-of-the-art work.

**Researches**	**Dataset**	**Proposed models**	**Optimal models' performance**
(Padma and Kumari 2020)	COVID chest Xray	2D CNN	Training ACC: 99% Validation ACC: 98.3%
(Nazish et al. 2021)	COVID-19 patients x-rays	Logistic Regression SVM	SVM(96%)
(Jangam et al. 2022)	Five different datasets	VGG19, Rest101, DenseNet169, and WideRestNet502	Ensemble Training : 99.5 % Testing : 99.1 %
(Yadlapalli et al. 2022)	COVID19-CT images	InceptionV3, ResNet50, VGG16, and InceptionV3	VGG16 : 89%
(Chakraborty et al. 2022)	10,040 samples	AlexNet, VGG, ResNet18, and DenseNet	Not specified but overall Accuracy:93.43 % Sensitivity:93.68 % Specificity:99.%0 F1-Score:93.0 %
(Chakraborty et al. 2022)	COVID-19 and Pneumonia x-rays	ResNet18, AlexNet, DenseNet, and VGG16	DenseNet (96.43%)
(Khan et al. 2022)	BIMCV- COVID19+	EfficientNetB1, NasNetMobile, and MobileNetV2	EfficientNetB1 (96.3%)
(Aggarwal et al. 2022)	Two datasets	MobileNetV2, Xception, ResNet50V2, DenseNet121, inceptionResNetV2, VGG19, NASNetMobile, and Inceptionv3	Dataset 1: DenseNet121 (97%) taset 2: MobileNetV2 (85%)
(Odeh et al. 2022)	COVID-19 radiography database	VGG16, ResNet50, InceptionV3, and DenseNet121	ResNet50 Training ACC: 99.99% Validation ACC: 99.50% Testing ACC: 99.44%
Present work	COVID-19 radiography dataset	RestNet18 AlexNet, VGG16 and EfficientNetB1	ResNet18 Training acc: 99.64% Validation acc: 99.56% Testing acc: 99.57% F1 score: 99.57 % Precision: 99.57 % Recall: 99.57 % ROC-AUC : 99.88 %

### 6.2 Potential clinical applications

Developing an accurate deep transfer learning model for medical image detection and classification is critical in general. As we specifically explored COVID-19 related cases in our study, real-world clinical settings should also benefit from the deployment and implementation of the best model obtained. Furthermore, highlighting and showcasing its potential to provide automatic predictions using x-ray image samples can be particularly valuable, especially in remote areas with limited medical resources.

**Figure 11:**
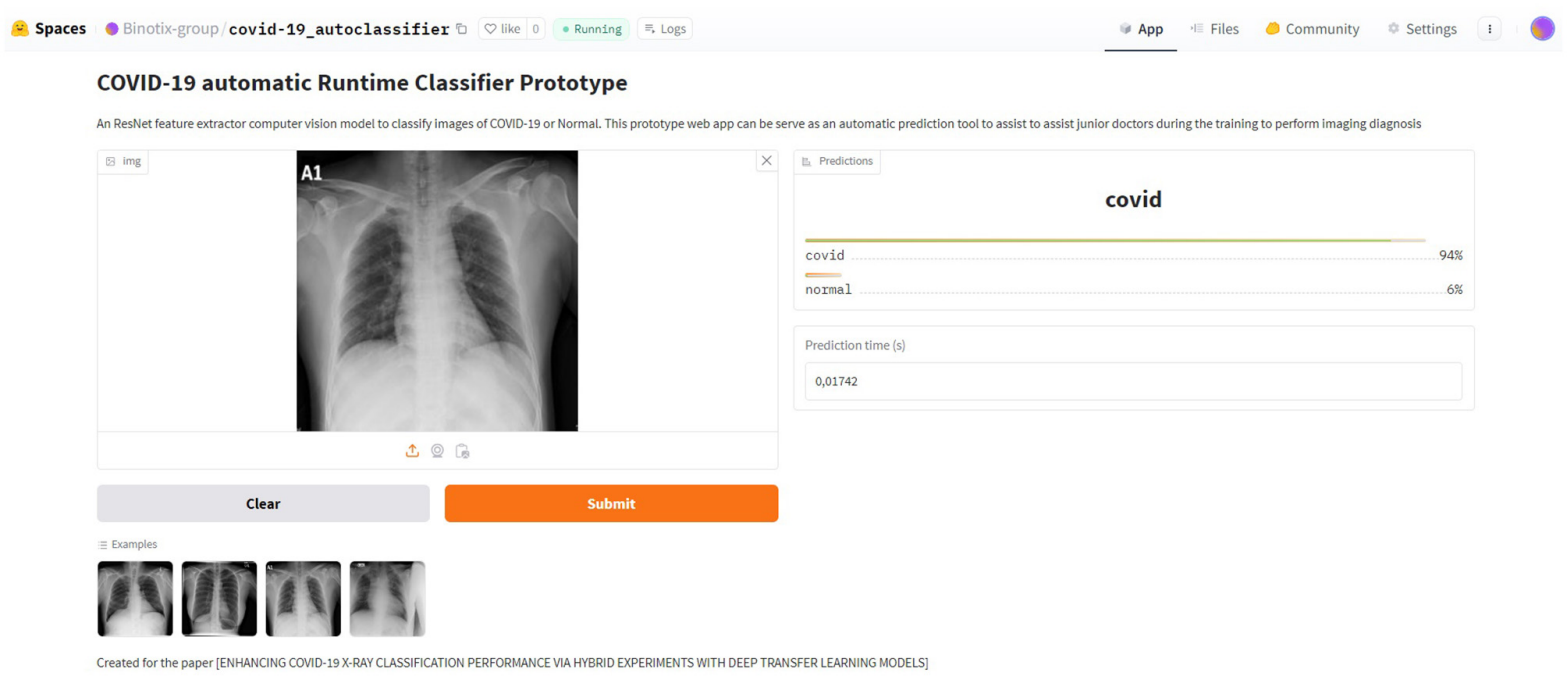
Visual interface of the Web application prototype for automatic detection.

In our study, we used Gradio to create an interactive interface for our deep transfer learning-based classifier for COVID-19 X-ray images. This interface, as shown in Figure 11, enabled us to prototype and test the deployment process efficiently, allowing domain experts such as medical junior doctors to interact with the model and provide valuable feedback.

Gradio is an open-source Python package that streamlines the development of user-friendly interfaces for machine learning models. It provides a user-friendly web-based interface to interact with ML models. It allows researchers to quickly generate a visual interface for their models, making it accessible to users, including those without a technical background, to interact with and interpret model outputs, which is especially valuable for clinical review. Gradio's ease of use and ability to share models via simple URLs significantly can enhance our collaboration with medical professionals. This is because Gradio enables real-time image classification using the deployed model. This approach not only improved the accessibility of our model but also ensured its robustness in real-world scenarios.

However, once fully deployed in production, our prototype can serve as a training aid tool for junior doctors, enhancing their diagnostic skills by providing a reliable reference for medical interpretation and helping them learn the patterns associated with COVID-19 on X-rays. This could improve their diagnostic confidence and accuracy, as they can compare their analyses with the model's predictions. We have launched a demo web application on the Hugging Face Spaces platform for testing purposes. The page of the application can be found at this link.

### 6.3 Limitations

As a limitation, Although the experimentations have been accomplished using the pro version of the Google Collaboratory, we were obliged to reduce the dataset size due to the long execution time (sometimes over 6 hours, not supported by the server). Moreover, the provided runtime GPU is limited per day and a limited number of sessions. The other limitation concerns the genetic algorithm parameters used. Due to the limited computation environment of the collab server, as mentioned earlier, the GA parameters have been defined in a small range to prevent ResourceExhausation error. The interval range of hyperparameter values is already discussed in the previous section.

Additionally, it is important to note that our study, while concentrated on image-based diagnosis in the context of COVID-19, does not include clinical information such as symptoms, signs, or laboratory tests. A comprehensive COVID-19 diagnosis often requires additional clinical data to inform treatment decisions accurately.

In future research, we will consider these dimensions and aim to integrate additional data sources, including clinical and imaging data. This approach may involve multimodal learning techniques to improve the diagnostic accuracy and applicability of new models as a more comprehensive diagnostic tool.

### 6.4 Academic and clinical value of the study beyond the pandemic

As previously mentioned in the literature review section, a multitude of studies pertaining to COVID-19 have been examined. Consequently, although the urgency for COVID-19 diagnosis commenced in 2019 and may have diminished following its peak period (2020–2022), during which it acquired significant academic and clinical attention, ongoing research remains pertinent. Recent research articles on COVID-19 have demonstrated significant impacts on analogous challenges. For example, (Kathamuthu et al. 2023) utilized convolutional neural networks (CNN) to analyze chest computed tomography images for COVID-19 screening purposes. (Agnihotri and Kohli 2023) conducted a study on the challenges, opportunities, and advancements in COVID-19 classification utilizing deep learning. (Misra et al. 2023) introduced a parallel ensemble transfer learning framework for COVID-19. Additionally, (Ali et al. 2024) developed a method for detecting COVID-19 pneumonia severity using deep learning algorithms and transfer learning.

These studies show the continued importance of COVID-19 detection and classification. Therefore, we believe that the framework we developed remains valuable for detecting respiratory and viral illnesses in general. This flexibility could future-proof the model for similar health challenges.

## 7 Conclusion and future recommendation

Transfer learning is a successful technique in the literature used to develop deep learning models for tasks related to the computer vision field. It provides the flexibility to build a new and robust model using other models already pre-trained on data related to the new domain rather than creating a new model from scratch. This research developed and improved distinct networks on the lung X-ray dataset to classify patients' scans as COVID-19 infected or normal and compare their performance to promote their utilization in medical diagnosis tasks. This study differs from previous works regarding different experimental process scenarios adopted to showcase the performance of each model, the modifications made to the models, various hyperparameter-based transfer learning processes, k-fold-based and the heuristic optimisation techniques such as GA-based hyperparameters tuning used to fine-tune the model's hyperparameters. To the best of our knowledge, these different experimentation scenarios have proven to assist in getting the best hyperparameters set for the best models with state-of-the-art results. Thus, as already revealed in the discussion section, the ResNet18 achieved the best test accuracy of 99.57% and 99.57 for F1-score, precision, recall and AUC average, which is better than the results accomplished in most recent works, including (Odeh et al. 2022); (Aggarwal et al. 2022).

In future work, we plan to extend the methodology implemented in this study to other pre-trained networks, including an exploration of some segmentation techniques (Zaitoun and Aqel, 2015), which could better improve the results. Furthermore, instead of YOLOv4, experimenting with the latest versions, such as YOLOv5 or V8 and detection transformers like DETR (Moustapha et al. 2023), should be considered as it is among the rising topics in the literature. ResNet18 can classify individual images better as COVID-19 or normal cases based on the results obtained and compared with previously achieved results. Finally, The best model of the study has the potential to achieve automatic predictions through the use of input images in a simulated web app, which has been deployed online as a prototype simulation; therefore, it can serve as an essential supplement for imaging diagnosis in remote areas with scarce medical resources and help in training junior doctors to perform imaging diagnosis.

## Data Availability

Publicly available datasets were analyzed in this study. This data can be found here: https://www.kaggle.com/datasets/asraf047/covid19-pneumonia-normal-chest-xray-pa-dataset.

## Supplementary material

Supplementary Figure 1 Normal chest X-ray sample.

Supplementary Figure 2 COVID-19 chest X-ray sample.
