# Supplementary figures and images for: Enhancing COVID-19 classification of X-ray images with hybrid deep transfer learning models
Source: Front Artif Intell. 2025 Oct 13;8:1646743. doi: 10.3389/frai.2025.1646743 (PMC12555394; doi:10.3389/frai.2025.1646743)

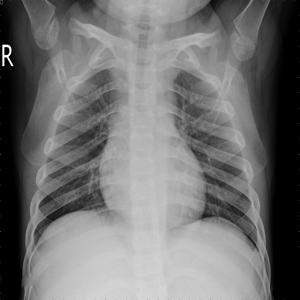

Supplement: Supplementary Figure 1 — Normal chest X-ray sample. [file Image_1.jpg]

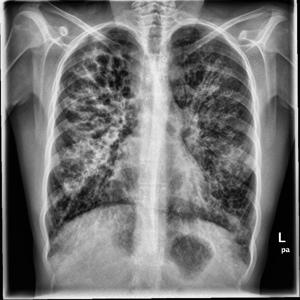

Supplement: Supplementary Figure 2 — COVID-19 chest X-ray sample. [file Image_2.jpg]
